# TNFAIP8 polarity proteins mediate T cell development by propagating lipid second messenger signaling in thymocytes

**DOI:** 10.1038/s41419-026-09100-6

**Published:** 2026-07-27

**Authors:** Jingtao Gao, Cun Guo, Yang Lv, Han Wang, Sheng Chen, Peili Zhuang, Miaomiao Song, Xueqin Tian, Shan Jiang, Wei Lu, Lu Zhang, Peiqing Zhao, Xiaohan Wu, Xiangfeng Song, Xiaohong Kang, Youhai H. Chen, Tingmin Chang, Hui Wang, Yunwei Lou

**Affiliations:** 1 Henan Health Commission Key Laboratory of Gastrointestinal Cancer Prevention and Treatment, Department of Gastroenterology, The First Affiliated Hospital of Henan Medical University (original name: Xinxiang Medical University), Xinxiang, China; 2 Henan International Joint Laboratory of Immunology and Model Animals, School of Medical Technology, Henan Medical University (original name: Xinxiang Medical University), Xinxiang, China; 3 Henan Collaborative Innovation Center of Molecular Diagnosis and Laboratory Medicine, School of Medical Technology, Henan Medical University (original name: Xinxiang Medical University), Xinxiang, China; 4 School of Basic Medical Sciences, Xinjiang Medical University, Urumqi, China; 5 Center of Translational Medicine, Zibo Central Hospital, Shandong University, Zibo, China; 6 School of Basic Medical Sciences, Henan Medical University (original name: Xinxiang Medical University), Xinxiang, China; 7 Department of Radiation Oncology, The First Affiliated Hospital of Henan Medical University (original name: Xinxiang Medical University), Xinxiang, China; 8 Faculty of Pharmaceutical Sciences, Shenzhen University of Advanced Technology, Shenzhen, China

**Keywords:** Lymphocyte differentiation, Lymphocyte activation

## Abstract

T lymphocytes are primarily generated in the thymus through a complex process of positive and negative selections. The molecular mechanisms underlying this process are not fully understood. Here, we report that TNFAIP8 (TNF-α-induced protein 8-like) family of polarity proteins mediates T cell development by propagating phosphoinositide signaling in the thymus. Genetic deletion of hematopoietic members of the TNFAIP8 family resulted in dramatic reductions in thymus size, cellularity, and corticomedullary differentiation. Total thymocyte counts in *Tnfaip8*-deficient mice were reduced by more than 50%, which was associated with marked reductions in T cell numbers in blood and in secondary lymphoid organs, as compared to wild-type mice. Bone marrow chimeric experiments revealed that the thymic developmental defect was hematopoietic cell-intrinsic. TNFAIP8 proteins controlled double negative (DN) thymocyte proliferation and metabolism during positive selection by promoting PI3K-AKT/mTOR signaling through phosphoinositide second messengers. Pharmaceutical activation of AKT partially rectified the thymic developmental defect of *Tnfaip8*-deficient mice. Thus, TNFAIP8 polarity proteins are essential for thymic development of T lymphocytes, and genetic or pharmaceutical manipulation of the TNFAIP8 pathway may be useful for controlling T lymphocyte-mediated immunity.

## Introduction

Thymus is a highly generative organ of T lymphocytes with ~100 million thymocytes produced daily in young adult mice [1]. The mass production of new thymocytes gives ample choices for subsequent positive and negative selections to yield mature T lymphocytes with proper repertoire for adaptive immune responses. Thymocyte development follows ordered progressive steps from CD4^-^CD8^-^ (double negative, DN, subdivided into stages DN1-DN4) through CD4^+^CD8^+^ (double positive, DP) to single positive (CD4^+^CD8^-^, CD4SP; or CD4^-^CD8^+^, CD8SP) stages which are driven by a complex interplay of signaling pathways [2]. CD4SP and CD8SP cells that survive selections must still undergo a series of steps for final maturation before they gain functional competency and enter the peripheral T cell pool [3]. During these processes, the precise control of thymocyte proliferation is essential [4]. The clonal expansion at this proliferative phase provides cellular basis for generating a highly diversified TCR repertoire and is also required for the differentiation of DN thymocytes to the DP stage [5, 6]. Although the crucial role of this proliferative burst has been established, the molecular program linking developmental signals to proliferative events remains to be fully elucidated.

The PI3K-AKT-mTOR pathway plays crucial roles in thymocyte development and peripheral T lymphocyte homeostasis. Genetic ablation of key signaling components of this pathway often results in a profound block in the transition of DN to DP thymocytes and a concomitant reduction in the expansion of thymocytes following β selection [7, 8]. Moreover, recent studies also have revealed a critical role of mTOR in early T lymphopoiesis [9, 10], T cell differentiation [11–13], quiescence, and survival under homeostatic condition [14]. On the other hand, aberrant activation of PI3K-AKT-mTOR pathway would result in unrestrained cell growth, survival, and proliferation, ultimately leading to T cell lymphoma or autoimmune diseases [15, 16]. Accordingly, stringent control of this pathway is required to secure normal thymocyte development and to avoid pathological consequences.

The TNFAIP8-like (tumor necrosis factor-α-induced protein 8-like) family of proteins consists of four members: TNFAIP8 (hereafter TIPE0), TIPE1 (TNFAIP8L1), TIPE2 (TNFAIP8L2) and TIPE3 (TNFAIP8L3) [17]. Among these, three members, i.e., TIPE0, TIPE1, and TIPE2, are constitutively expressed at high levels in hematopoietic cells whereas TIPE3 is absent in most immune cells [18]. Previous reports have demonstrated that all TNFAIP8 proteins can bind phosphoinositide second messengers such as phosphatidylinositol 4,5-bisphosphate (PtdIns(4,5)P2, aka PIP2) and phosphatidylinositol 3,4,5-trisphosphate (PtdIns(3,4,5)P3, aka PIP3), serving as lipid second messenger-transfer proteins [19–22]. Recently, we demonstrated that TIPE0 and TIPE1 play redundant roles in regulating specific cellular processes [23]. In this study, we asked whether the TNFAIP8 family plays a role in thymic development and differentiation using both in vitro and in vivo experimental approaches, and discovered that TNFAIP8 proteins mediate T cell development by propagating lipid second messenger signaling in thymocytes.

## Results

### TNFAIP8 family proteins are required for T cell development in the thymus

To understand the physiological roles of TNFAIP8 family proteins in T cell development, we first evaluated the effects of genetic deletion of the *Tipe0*, *Tipe1* or *Tipe2* genes. Consistent with previous reports [23–25], no significant defects in the percentages or absolute numbers of DN, DP, CD4SP, and CD8SP thymocytes were found in *Tipe0*^*-/-*^, *Tipe1*^*-/-*^ or *Tipe2*^*-/-*^ mice (Fig. S1A, B). To determine whether *Tipe* genes played a redundant role, we analyzed mice deficient in two of the *Tipe* genes and found that total cell numbers were not altered substantially in the thymuses of these mice compared with their wild-type (WT) counterparts (Fig. S1C). To further investigate the potential functional redundancy of TNFAIP8 proteins, we generated mice with germline deletions of all three hematopoietic *Tipe* genes (*Tipe0*^*-/-*^*Tipe1*^*-/-*^*Tipe2*^*-/-*^ mice) by serially crossing strains of aforementioned double knockout mice (Fig. S2A-D).

**Fig. 1:**
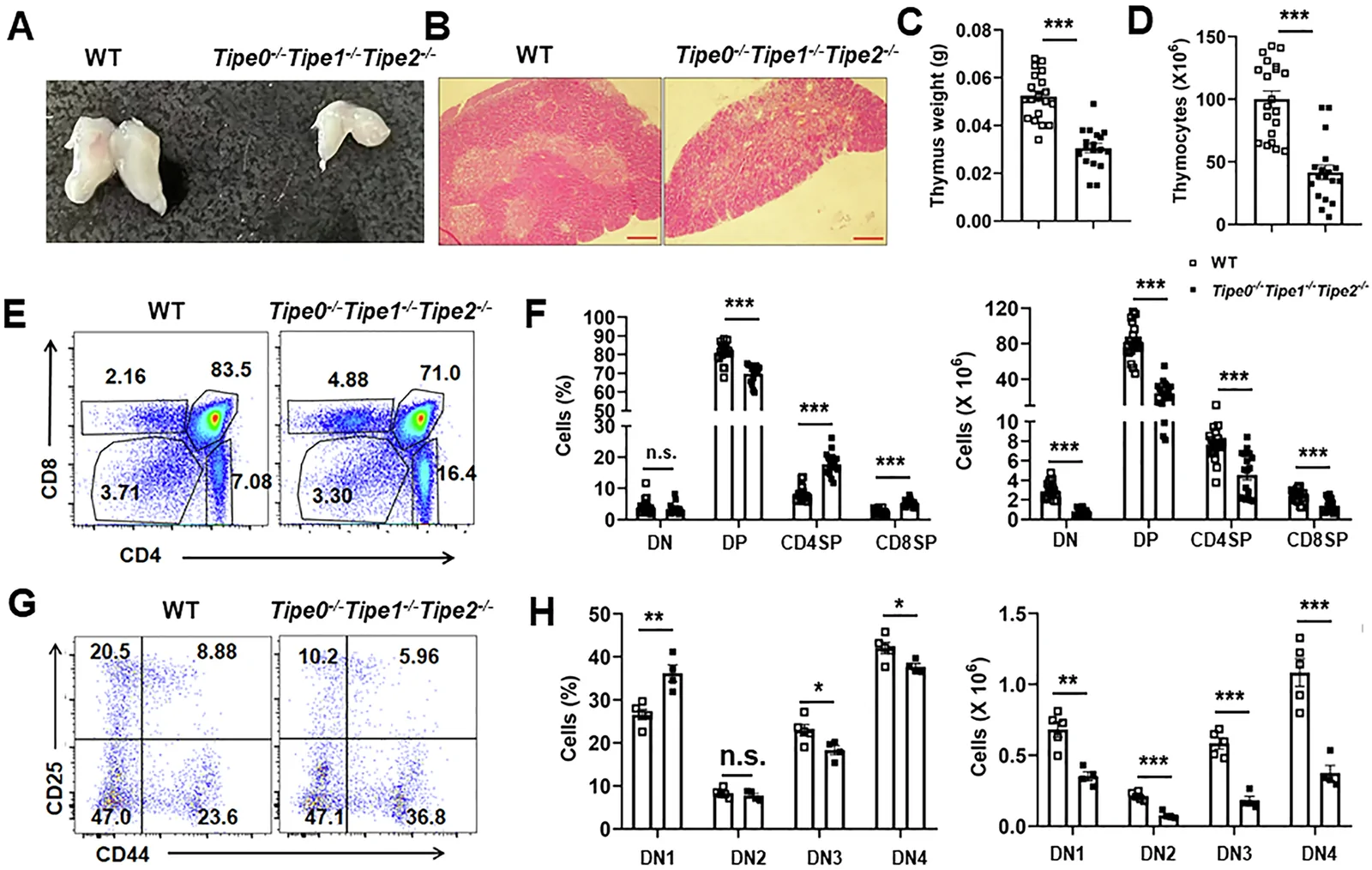
Defective T lymphopoiesis in *Tipe0*^*-/-*^*Tipe1*^*-/-*^*Tipe2*^*-/-*^ mice. **A** Thymus images of 8-week-old WT and *Tipe0*^*-/-*^*Tipe1*^*-/-*^*Tipe2*^*-/-*^ mice. **B** Haematoxylin and eosin staining of thymus sections from WT and *Tipe0*^*-/-*^*Tipe1*^*-/-*^*Tipe2*^*-/-*^ mice; the cortex (darker) and medulla (lighter) can be distinguished by the intensity of staining. Scale bars, 100 μm; original magnification х4. Thymus weight (**C**) and total cell numbers of thymocytes (**D**) from WT (*n* = 20) and *Tipe0*^*-/-*^*Tipe1*^*-/-*^*Tipe2*^*-/-*^ (*n* = 18) mice. Data are pooled from five independent experiments. FACS analysis of thymic subpopulations based on surface staining of CD4 and CD8 from WT (n = 20) and *Tipe0*^*-/-*^*Tipe1*^*-/-*^*Tipe2*^*-/-*^ (*n* = 18) mice, presented as a representative FACS plot (**E**) and summary graph (**F**). Numbers in or adjacent to outlined areas indicate percent CD8^+^CD4^-^ cells (top left), CD8^+^CD4^+^ cells (top right), CD8^-^CD4^-^ cells (bottom left) or CD8^-^CD4^+^ cells (bottom right). Data are pooled from five independent experiments. FACS analysis of CD8^-^CD4^-^ DN thymocytes based on expression of CD25 and CD44 from WT (*n* = 5) and *Tipe0*^*-/-*^*Tipe1*^*-/-*^*Tipe2*^*-/-*^ (*n* = 4) mice, presented as a representative FACS plot (**G**) and summary graph of DN1-DN4 stages (**H**). DN1: CD44^+^CD25^-^; DN2: CD44^+^CD25^+^; DN3: CD44^-^CD25^+^; DN4: CD44^-^CD25^-^. Data are representative of five independent experiments with at least three mice per group. Data are presented as mean ± SEM. Student’s *t* test (**C**, **D**, **F** and **H**), *P < 0.05, **P < 0.01, ***P < 0.001, n.s. non-significant.

Examination of thymuses revealed that the thymic architecture was considerably altered in *Tipe0*^*-/-*^*Tipe1*^*-/-*^*Tipe2*^*-/-*^ mice, as characterized by much smaller areas of the cortex, resulting in thymuses that were much smaller than those of WT control animals (Fig. 1A, B). Moreover, there was a clear lack of compartmentalization of the thymus, i.e., cortex *vs*. medulla, as well as a loss of cellularity in *Tipe0*^*-/-*^*Tipe1*^*-/-*^*Tipe2*^*-/-*^ mice (Fig. 1B). Indeed, *Tipe0*^*-/-*^*Tipe1*^*-/-*^*Tipe2*^*-/-*^ mice exhibited markedly reduced thymic weight and cell numbers (Fig. 1C, D). A subtle but statistically significant decrease in DP percentage and increase in CD4SP and CD8SP percentages were observed in *Tipe0*^*-/-*^*Tipe1*^*-/-*^*Tipe2*^*-/-*^ mice (Fig. 1E, F). Nevertheless, total cell numbers in each major thymocyte subset were markedly reduced in *Tipe0*^*-/-*^*Tipe1*^*-/-*^*Tipe2*^*-/-*^ animals (Fig. 1F, right panel). Furthermore, the percentage of DN1 was increased, whereas percentages of DN3 and DN4 were decreased in *Tipe0*^*-/-*^*Tipe1*^*-/-*^*Tipe2*^*-/-*^ animals (Fig. 1G, H). However, due to the overall reduction of thymic cellularity, the numbers of all DN subsets were significantly reduced (Fig. 1H, right panel). The *Tipe0*^*-/-*^*Tipe1*^*-/-*^*Tipe2*^*-/-*^ mice had a reduced number of thymic γδ T cells, whereas the numbers of invariant NKT (iNKT) cells and regulatory T (Treg) cells were comparable between WT and *Tipe0*^*-/-*^*Tipe1*^*-/-*^*Tipe2*^*-/-*^ mice (Fig. S3). Collectively, these results indicate that TNFAIP8 family of proteins plays redundant roles in T cell development, and loss of one can be adequately compensated for by other TNFAIP8 proteins.

### Thymic positive selection requires TNFAIP8

**Fig. 2:**
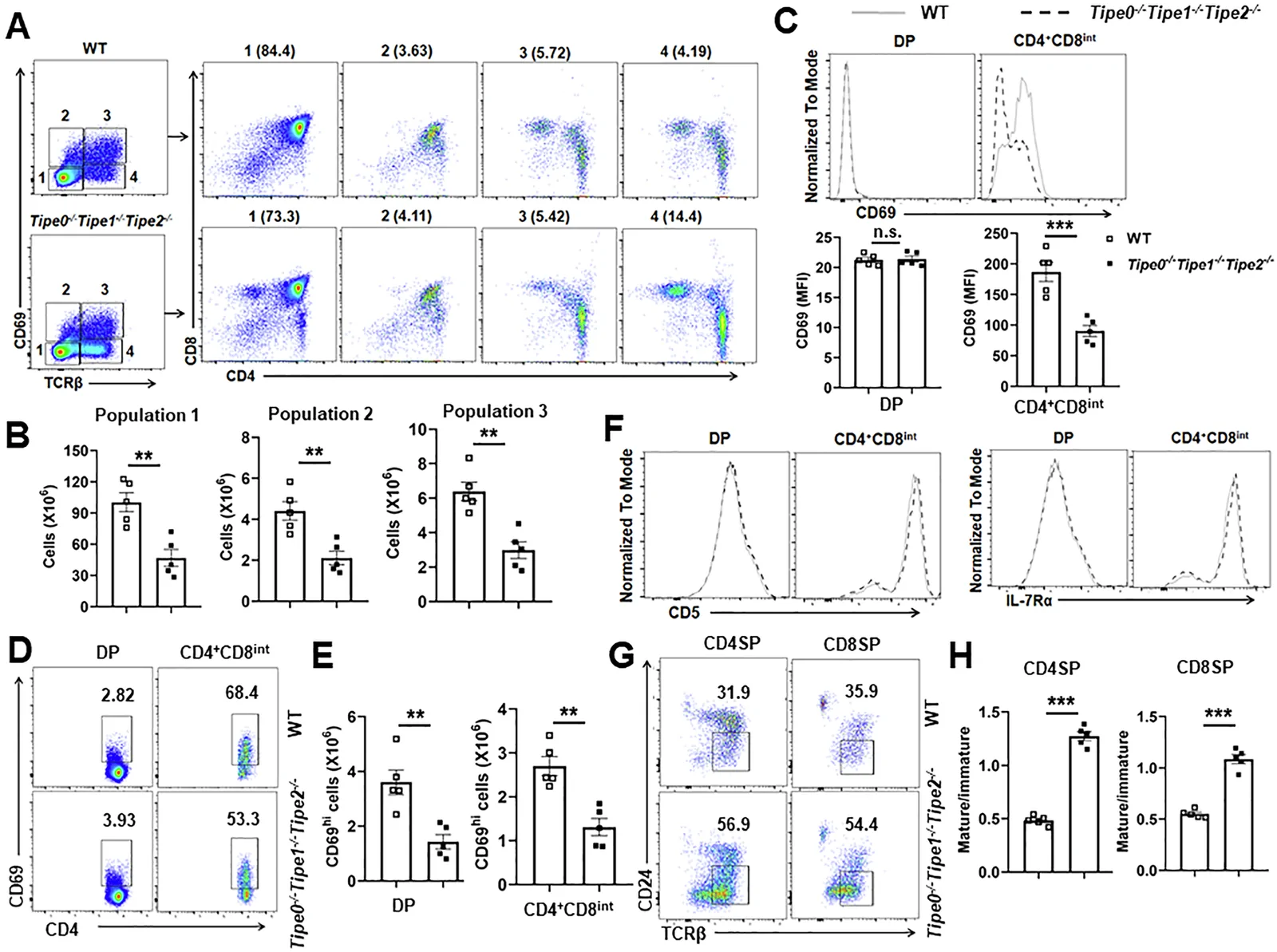
Impaired thymic positive selection in *Tipe0*^*-/-*^*Tipe1*^*-/-*^*Tipe2*^*-/-*^ mice. **A** FACS analysis of the surface expression of CD69 and TCRβ on thymocytes from WT (*n* = 5) and *Tipe0*^*-/-*^*Tipe1*^*-/-*^*Tipe2*^*-/-*^ (*n* = 5) mice, showing four subpopulations of thymocytes. Population 1 (preselection DP thymocytes): TCRβ^lo^CD69^lo^; population 2 (a transitional population initiating positive selection): TCRβ^int^CD69^hi^; population 3 (a population immediately after positive selection): TCRβ^hi^CD69^hi^; population 4 (a more mature postselection SP thymocyte population): TCRβ^hi^CD69^lo^. Numbers in outlined areas at left indicate subpopulations gated at right, and numbers in parentheses above plots at right indicate percent thymocytes in each subpopulation. **B** Summary graph of the thymocyte subpopulations from (**A**). **C** Surface staining of CD69 on gated DP or CD4^+^CD8^int^ thymocytes from WT (*n* = 5) and *Tipe0*^*-/-*^*Tipe1*^*-/-*^*Tipe2*^*-/-*^ (*n* = 5) mice. Below, mean fluorescence intensity (MFI) of surface CD69 staining on gated DP or CD4^+^CD8^int^ cells. FACS analysis of CD69 expression on DP or CD4^+^CD8^int^ cells, as shown of percentage (**D**) and absolute number (**E**). **F** Surface staining of CD5 and IL-7Rα on gated DP or CD4^+^CD8^int^ cells from WT (*n* = 5) and *Tipe0*^*-/-*^*Tipe1*^*-/-*^*Tipe2*^*-/-*^ (*n* = 5) mice. Surface staining of CD24 and TCRβ on gated CD4SP or CD8SP thymocytes from WT (*n* = 5) and *Tipe0*^*-/-*^*Tipe1*^*-/-*^*Tipe2*^*-/-*^ (*n* = 5) mice. Numbers adjacent to outlined areas indicate percent cells in the CD24^lo^TCRβ^hi^ gate (**G**). Ratio of mature (CD24^neg-lo^) cells to immature (CD24^hi^) cells (Mature/immature) (**H**). Data are representative of three independent experiments. Data are presented as mean ± SEM. Student’s *t* test (**B**, **C**, **E** and **H**), **P < 0.01, ***P < 0.001, n.s. non-significant.

To precisely define the specific developmental stage at which thymocytes were interrupted in *Tipe0*^*-/-*^*Tipe1*^*-/-*^*Tipe2*^*-/-*^ mice, we quantified cells at four distinct developmental stages determined by the expression of TCRβ and CD69. This method could separate thymocytes into four populations (Fig. 2A) [26, 27]. Because of the drastic decrease of total thymocytes in *Tipe0*^*-/-*^*Tipe1*^*-/-*^*Tipe2*^*-/-*^ mice, the absolute numbers of population 1, population 2, and population 3 were all decreased (Fig. 2B). We found that CD69 expression, a critical maker of successful positive selection, in DP cells was comparable between these two groups, whereas CD69 expression in CD4^+^CD8^int^ cells was markedly downregulated as compared with WT controls (Fig. 2C). Further analysis revealed a substantially reduced absolute numbers of CD69^hi^ cells within the total DP thymocyte population and the CD4^+^CD8^int^ transitional thymocyte population of *Tipe0*^*-/-*^*Tipe1*^*-/-*^*Tipe2*^*-/-*^ mice (Fig. 2D, E). These results suggest a defect of the *Tipe0*^*-/-*^*Tipe1*^*-/-*^*Tipe2*^*-/-*^ thymocytes in positive selection.

We also found that surface expression of survival receptor IL-7R was comparable in DP and CD4^+^CD8^int^ thymocytes from two groups (Fig. 2F). Similar results were obtained in the analysis of surface expression of the adhesion molecule CD5, another positive selection maker, in DP and CD4^+^CD8^int^ thymocytes (Fig. 2F). However, the maturation of CD4^+^ or CD8^+^ SP was considerably enhanced in *Tipe0*^*-/-*^*Tipe1*^*-/-*^*Tipe2*^*-/-*^ mice, as shown by the higher ratio of mature (CD24^lo^) TCRβ^hi^CD4^+^ or TCRβ^hi^CD8^+^ thymocytes to immature (CD24^hi^) TCRβ^hi^CD4^+^ or TCRβ^hi^CD8^+^ thymocytes, respectively (Fig. 2G, H). These findings suggest that there might be a homeostatic mechanism to compensate for the thymic T lineage developmental defects in *Tipe0*^*-/-*^*Tipe1*^*-/-*^*Tipe2*^*-/-*^ mice.

### Markedly reduced peripheral T cell numbers in *Tipe0*^*-/-*^*Tipe1*^*-/-*^*Tipe2*^*-/-*^ mice

**Fig. 3:**
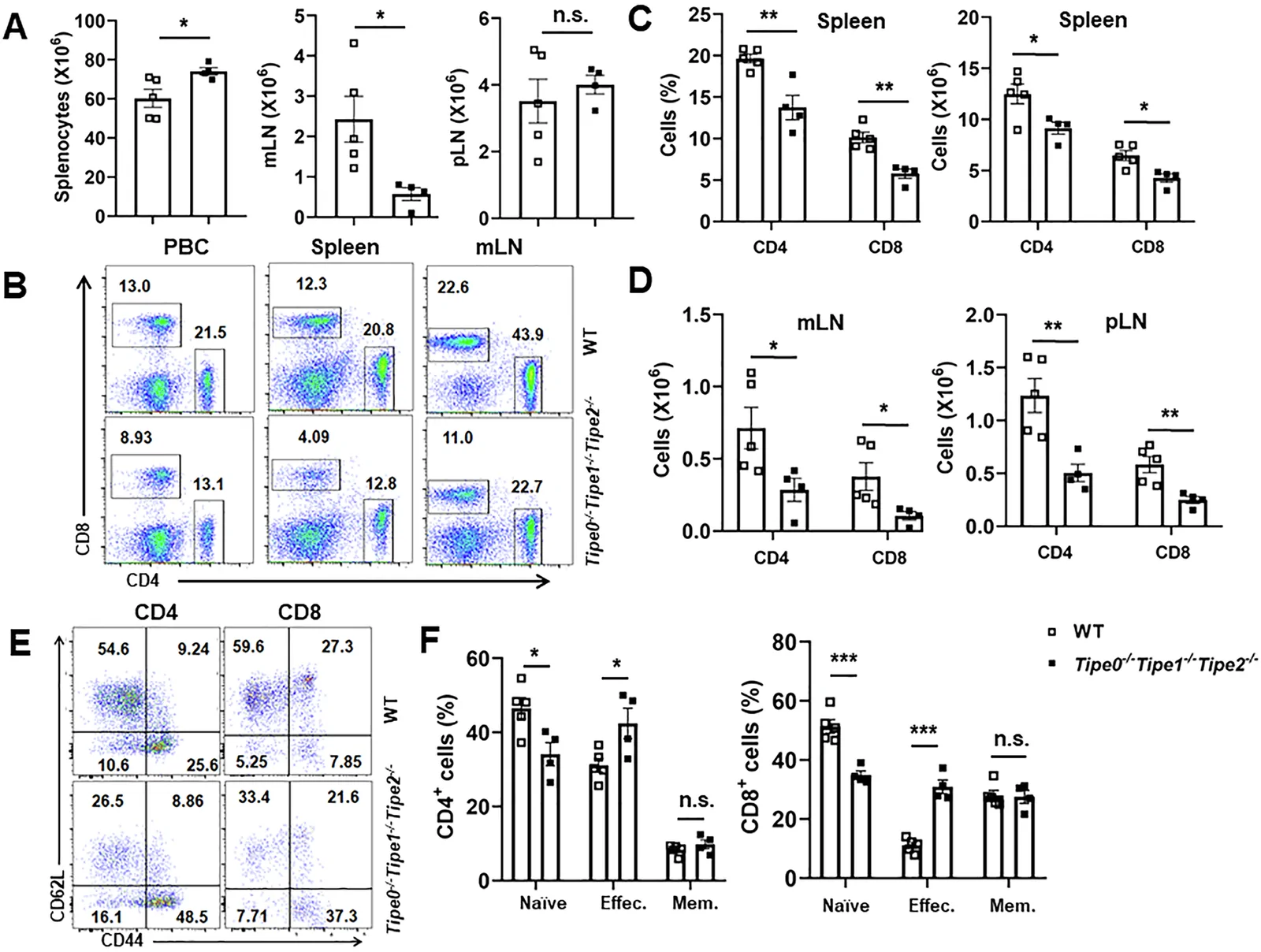
Lymphopenia in *Tipe0*^*-/-*^*Tipe1*^*-/-*^*Tipe2*^*-/-*^ mice. **A** Total cell numbers in the spleen, mLN and pLN from WT (*n* = 5) and *Tipe0*^*-/-*^*Tipe1*^*-/-*^*Tipe2*^*-/-*^ (*n* = 4) mice. Analysis of peritoneal T cells. Representative plots show that the CD4^+^ and CD8^+^ T cells in PBC, spleen and mLN (**B**). Frequency and numbers of CD4^+^ and CD8^+^ T cells from indicated subsets in (**B**) are shown accordingly (**C**, **D**). FACS profiles (**E**) and quantification of percentages of splenic naïve CD4^+^ and CD8^+^ T cells (CD62L^hi^CD44^lo^), effector CD4^+^ and CD8^+^ T cells (CD62L^lo^CD44^hi^) and memory CD4^+^ and CD8^+^ T cells (CD62L^hi^CD44^hi^) from WT (*n* = 5) and *Tipe0*^*-/-*^*Tipe1*^*-/-*^*Tipe2*^*-/-*^ (*n* = 4) mice. Data are representative of three independent experiments. Data are presented as mean ± SEM. Student’s *t* test (**C**, **D** and **F**), *P < 0.05, **P < 0.01, ***P < 0.001, n.s. non-significant.

We next investigated whether the peripheral T cell pool was affected in *Tipe0*^*-/-*^*Tipe1*^*-/-*^*Tipe2*^*-/-*^ mice. The spleen exhibited normal size, although total cell numbers of spleen were slightly increased in *Tipe0*^*-/-*^*Tipe1*^*-/-*^*Tipe2*^*-/-*^ mice (Fig. 3A). Compared to WT mice, *Tipe0*^*-/-*^*Tipe1*^*-/-*^*Tipe2*^*-/-*^ mice had significantly fewer cells in the mesenteric lymph nodes (mLN), but a comparable number of cells in the peripheral inguinal lymph nodes (pLN) (Fig. 3A). However, the total numbers of CD4^+^ and CD8^+^ T cells in peripheral blood cells (PBC), spleen, mLN, and pLN were all remarkably reduced in *Tipe0*^*-/-*^*Tipe1*^*-/-*^*Tipe2*^*-/-*^ mice (Fig. 3B–D). Interestingly, among the conventional CD4^+^ and CD8^+^ T cell compartments, *Tipe0*^*-/-*^*Tipe1*^*-/-*^*Tipe2*^*-/-*^ mice had an expanded CD62L^lo^CD44^hi^ population with an activated phenotype, with a correspondingly lower percentage of CD62L^hi^CD44^lo^ naïve T cells (Fig. 3E, F). Collectively, these data showed that deficiency in TNFAIP8 not only led to fewer mature T cells in the periphery but also resulted in a phenotype for *Tipe0*^*-/-*^*Tipe1*^*-/-*^*Tipe2*^*-/-*^ T cells reminiscent of that of effector memory cells, a frequent observation in partially lymphopenic mice that reflects “homeostatic” expansion of T cell populations.

### TNFAIP8 regulates T cell development in a cell-intrinsic manner

**Fig. 4:**
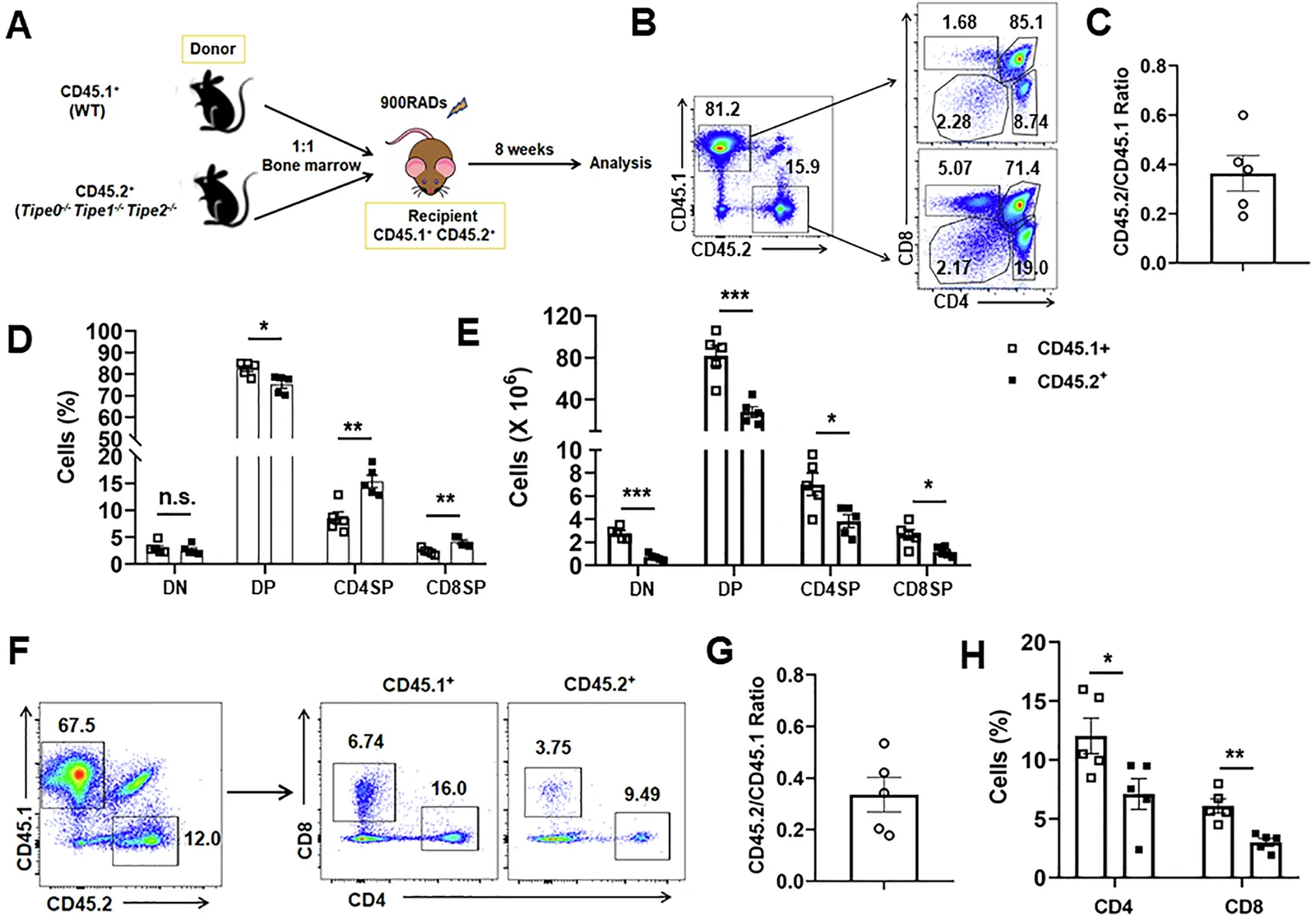
Cell-intrinsic defect of *Tipe0*^*-/-*^*Tipe1*^*-/-*^*Tipe2*^*-/-*^ thymocyte in development. **A** Schematic of experimental design. WT recipients (CD45.1^+^/CD45.2^+^, *n* = 5) were lethally irradiated, and after 3 h, transferred with 1:1 mixed bone marrow cells from WT (CD45.1^+^) and *Tipe0*^*-/-*^*Tipe1*^*-/-*^*Tipe2*^*-/-*^ (CD45.2^+^) mice. Eight weeks after the transfer, thymocytes of recipient mice at different developmental stage were identified by congenic CD45.1 and CD45.2 markers. **B** Representative FACS plots showing CD45.1 and CD45.2 staining in thymocytes (left panel) and CD4 and CD8 staining of live gated thymocytes (right panel). **C** Scatter plots showing ratios of CD45.2^+^ to CD45.1^+^ thymocytes. **D**, **E** Frequencies and cell numbers of indicated subsets as in (**B**) is shown accordingly (*n* = 5). **F** Representative FACS plots showing CD45.1 and CD45.2 staining in splenocytes (left panel) and CD4 and CD8 staining of live gated splenocytes (right panel). **G** Scatter plots showing ratios of CD45.2^+^ to CD45.1^+^ splenocytes. **H** Frequencies of splenic CD4^+^ and CD8^+^ T cells as in (**F**) is shown accordingly (*n* = 5). Data are representative of two independent experiments. Data are presented as mean ± SEM. Student’s *t* test (**D**, **E** and **H**), *P < 0.05, **P < 0.01, ***P < 0.001, n.s. non-significant.

To examine whether the thymocyte development defect of *Tipe0*^*-/-*^*Tipe1*^*-/-*^*Tipe2*^*-/-*^ mice was cell intrinsic, we performed a competitive BM repopulation study (Fig. 4A) [28]. Eight weeks after the cell transfer, repopulated thymuses were analyzed by flow cytometry using CD45.1 and CD45.2 congenic makers (Fig. 4B). Under the identical environment from recipients, *Tipe0*^*-/-*^*Tipe1*^*-/-*^*Tipe2*^*-/-*^ (CD45.2^+^) BM-derived thymocytes phenocopied the defect of the *Tipe0*^*-/-*^*Tipe1*^*-/-*^*Tipe2*^*-/-*^ mice, exhibiting a decrease in the frequency of DP cells and an increase in percentages of CD4SP and CD8SP cells (Fig. 4B). However, the fitness of WT T lineage cells within the thymi was greater than that of *Tnfaip8*-deficient counterparts. The CD45.2^+^ TNFAIP8 KO to CD45.1^+^ WT ratios for thymocytes were drastically decreased to 0.35:1 (Fig. 4C). Consistently, *Tipe0*^*-/-*^*Tipe1*^*-/-*^*Tipe2*^*-/-*^ donor-derived thymocytes in chimeric mice exhibited significant developmental defects similar to those observed in *Tipe0*^*-/-*^*Tipe1*^*-/-*^*Tipe2*^*-/-*^ mice (Fig. 4D, E). Moreover, consistent with severe defects in thymocytes generation, in the spleens of the recipient mice, there were significantly less *Tipe0*^*-/-*^*Tipe1*^*-/-*^*Tipe2*^*-/-*^ T cells than WT T cells in both CD4 and CD8 compartments (Fig. 4F–H). Thus, these data suggest a cell-intrinsic role for TNFAIP8 in regulating thymocyte development.

### TNFAIP8 promotes the proliferation and metabolism of thymocytes

**Fig. 5:**
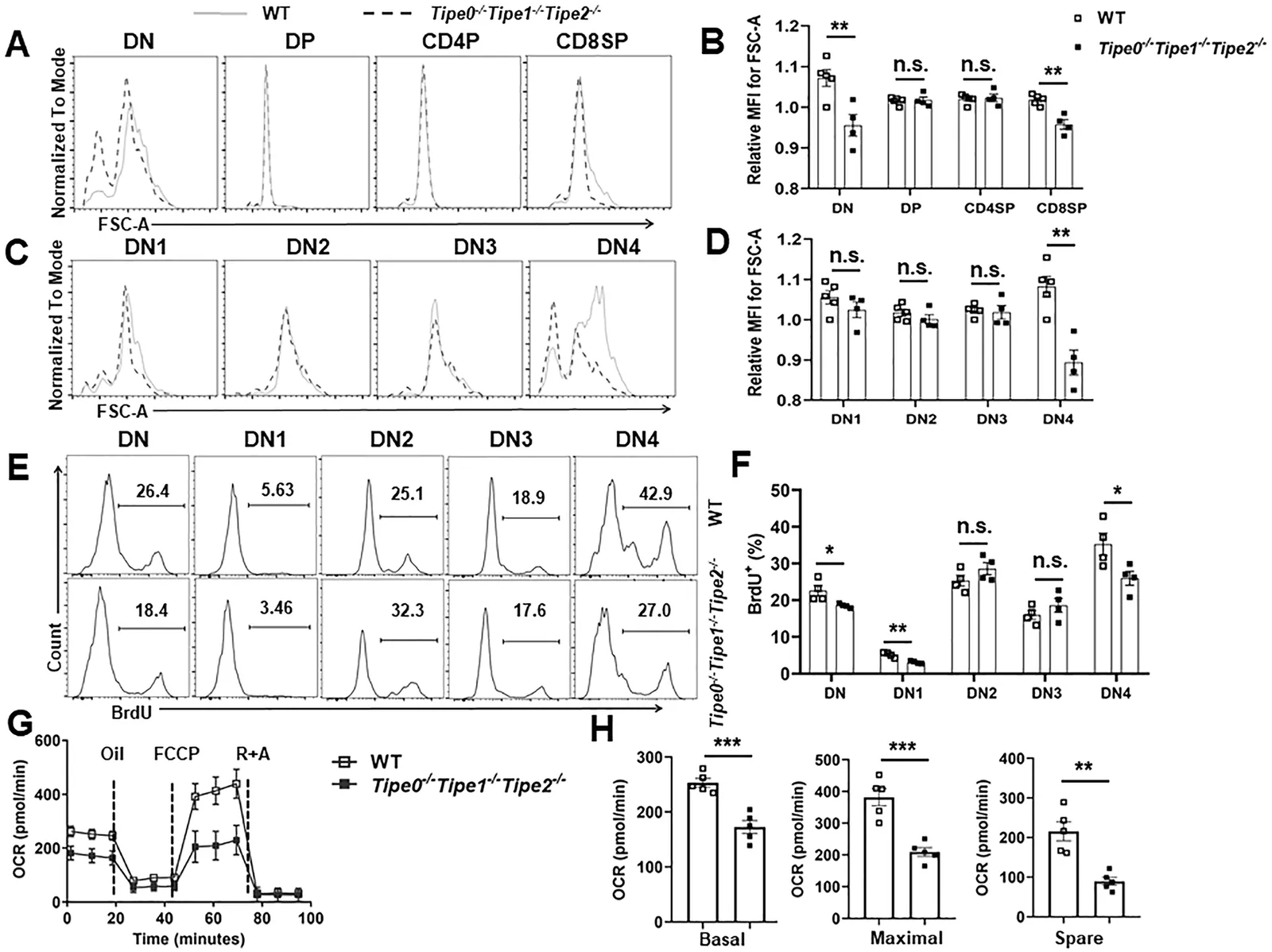
Impaired DN thymocyte proliferation and metabolism in *Tipe0*^*-/-*^*Tipe1*^*-/-*^*Tipe2*^*-/-*^ mice. **A** Representative histograms of forward scatters of area (FSC-A) of DN, DP, CD4SP and CD8SP from WT (*n* = 5) and *Tipe0*^*-/-*^*Tipe1*^*-/-*^*Tipe2*^*-/-*^ (*n* = 4) mice. **B** Means and SEM of relative cell sizes as in (**A**) are shown. The mean FSC-A of WT mice is artificially defined as 1.0. **C** Representative histograms of forward scatters of area (FSC-A) of DN1, DN2, DN3 and DN4 from WT (*n* = 5) and *Tipe0*^*-/-*^*Tipe1*^*-/-*^*Tipe2*^*-/-*^ (*n* = 4) mice. **D** Means and SEM of relative cell sizes as in (**C**) are shown. **E** Representative profiles of BrdU incorporation in DN thymocytes from WT (*n* = 5) and *Tipe0*^*-/-*^*Tipe1*^*-/-*^*Tipe2*^*-/-*^ (*n* = 4) mice at 4 h after BrdU injection. **F** Frequencies of BrdU^+^ cells among DN1-DN4 subsets as in (**E**). **G** Extracellular flux analysis of the oxygen consumption rates (OCRs) of DN thymocytes purified from WT (*n* = 5) and *Tipe0*^*-/-*^*Tipe1*^*-/-*^*Tipe2*^*-/-*^ (*n* = 5) mice under basal conditions or at maximum with the addition of oligomycin (Oli), the mitochondrial uncoupler carbonyl cyanide-4-(trifluoromethoxy) phenylhydrazone (FCCP), and rotenone plus antimycin A (R + A). **H** Summary graph of OCRs, including basal respiration, maximal respiration, and spare respiration capacity. Data are representative of three independent experiments. Data are presented as mean ± SEM. Student’s *t* test (**B**, **D**, **F** and **H**), *P < 0.05, **P < 0.01, ***P < 0.001, n.s. non-significant.

Previous reports have established that normal thymic cellularity is increased through massive proliferation of DN thymocytes [1, 29]. To examine the cellular basis for the reduced thymic cellularity in *Tipe0*^*-/-*^*Tipe1*^*-/-*^*Tipe2*^*-/-*^ mice, we first measured cell size (determined by FSC) and found that the effect of TNFAIP8 deletion on cell size was most profound on DN (Fig. 5A, B). Further analysis showed that, among DN subsets, remarkable reduction of cell size was observed in DN4 (Fig. 5C, D). The overall size of thymus is also dependent on thymocyte survival. We therefore measured cell survival by annexin V staining and found that comparable rate of cell death was observed in both WT and *Tnfaip8*-deficient DN, DP, CD4SP, and CD8SP thymocytes (Fig. S4A, B). After a short-term pulse, DN thymocytes (especially DN4 cells) of WT mice showed a highly proliferative profile (Fig. 5E) by BrdU incorporation. In contrast, the frequency of BrdU^+^ cells in DN subset (especially DN4 cells) was reduced in *Tipe0*^*-/-*^*Tipe1*^*-/-*^*Tipe2*^*-/-*^ mice (Fig. 5F). Consistent with DN subset, both CD4SP and CD8SP in *Tipe0*^*-/-*^*Tipe1*^*-/-*^*Tipe2*^*-/-*^ mice also had less uptake of BrdU in vivo (Fig. S4C, D). The proliferative defect in DN subset was further confirmed by endogenous Ki67 staining (Fig. S4E, F). Additionally, the oxidative phosphorylation (OXPHOS) rates both at baseline and at peak capacity in *Tnfaip8*-deficient DN cells were lower than those of WT DN cells (Fig. 5G, H). We infer that TNFAIP8 contributes to the production of a full T cell complement by promoting thymocyte proliferation and metabolism to establish normal thymocyte numbers.

### TNFAIP8 promotes DN cell metabolic programming and PI3K-AKT/mTOR activation

**Fig. 6:**
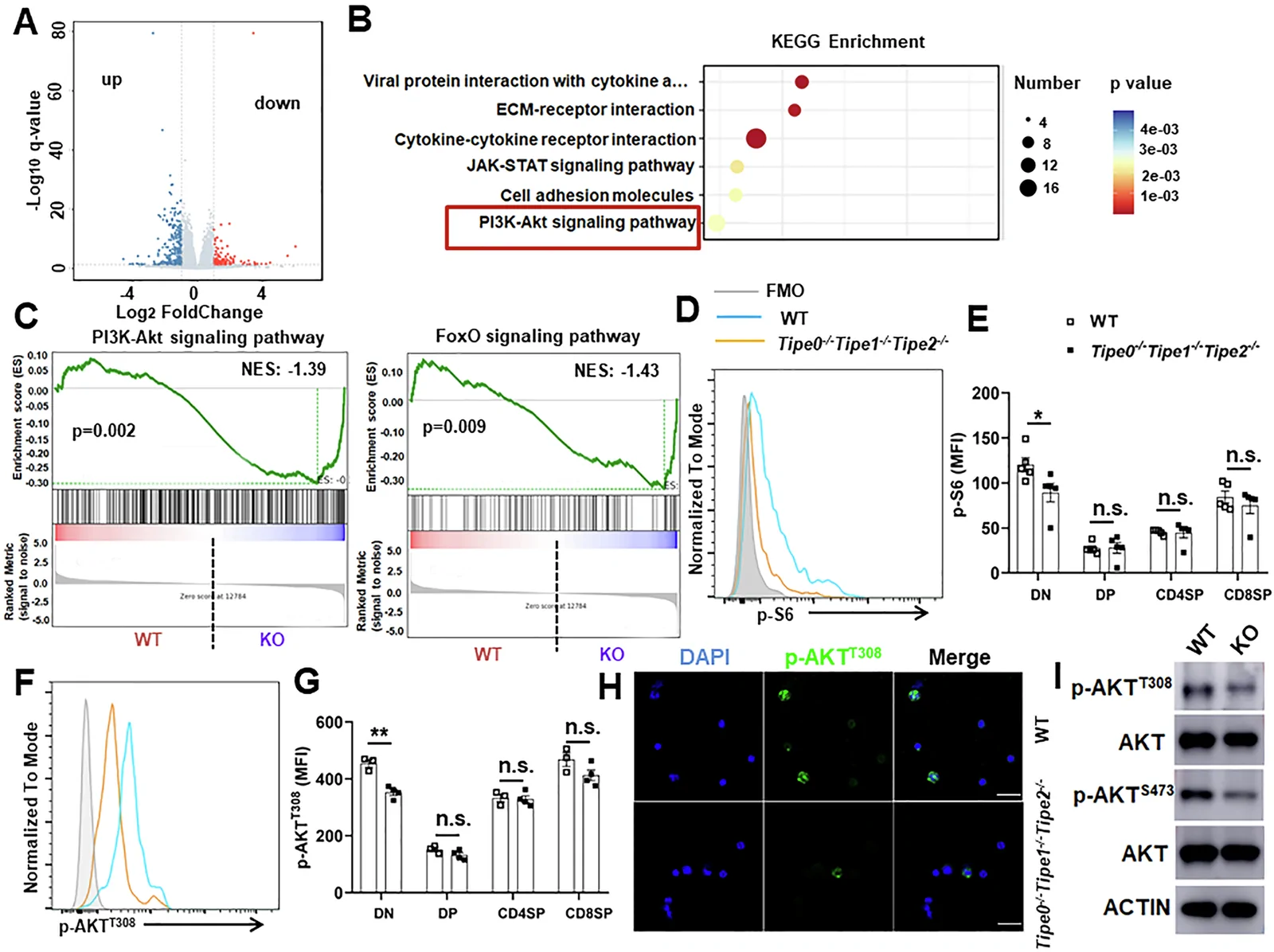
TNFAIP8 promotes PI3K-mTOR activation in DN thymocytes. **A** Volcano plot comparing global gene expression profiles between WT (*n* = 5) and *Tipe0*^*-/-*^*Tipe1*^*-/-*^*Tipe2*^*-/-*^ (*n* = 5) purified DN thymocytes. The red dots represent the transcripts with decreasing expression values (128 genes), whereas the blue dots signify transcripts with increasing expression values (271 genes) in *Tipe0*^*-/-*^*Tipe1*^*-/-*^*Tipe2*^*-/-*^ cells. **B** Kyoto Encyclopedia of Genes and Genomes (KEGG) enrichment analysis of upregulated and downregulated genes sets in *Tipe0*^*-/-*^*Tipe1*^*-/-*^*Tipe2*^*-/-*^ DN thymocytes compared to WT DN thymocytes. The top enriched KEGG terms shown were ranked by *p*-value. **C** GSEA reveals the PI3K-AKT-FoxO pathway as one of the most extensively downregulated pathways in *Tipe0*^*-/-*^*Tipe1*^*-/-*^*Tipe2*^*-/-*^ DN thymocytes. FACS analysis of phosphorylated (p-)S6 (Ser235/236) in DN, DP, CD4SP, and CD8SP from WT (*n* = 5) and *Tipe0*^*-/-*^*Tipe1*^*-/-*^*Tipe2*^*-/-*^ (*n* = 5) mice, presented as representative plots (**D**) only for DN and summary graph (**E**) for DN, DP, CD4SP, and CD8SP. FACS analysis of p-AKT(T308) in DN, DP, CD4SP, and CD8SP from WT (*n* = 3) and *Tipe0*^*-/-*^*Tipe1*^*-/-*^*Tipe2*^*-/-*^ (*n* = 3) mice, presented as representative plots (**F**) only for DN and summary graph (**G**) for DN, DP, CD4SP, and CD8SP. **H** Confocal microscopy of p-AKT(T308), as well as the DNA-binding dye DAPI (colors of labels above images indicate color of molecule (or dye) in image), in WT and *Tipe0*^*-/-*^*Tipe1*^*-/-*^*Tipe2*^*-/-*^ DN thymocytes. Scale bars, 100 μm; original magnification ×4. **I** Western blot analysis of the indicated proteins in WT and *Tipe0*^*-/-*^*Tipe1*^*-/-*^*Tipe2*^*-/-*^ DN thymocytes. Data are representative of three independent experiments. Data are presented as mean ± SEM. Student’s *t* test (**E** and **G**), *P < 0.05, **P < 0.01, n.s. non-significant.

To further explore the mechanisms by which TNFAIP8 regulates T lymphopoiesis, we performed RNA-seq analysis (Fig. 6A). Interestingly, KEGG analysis showed that one of the top pathways differentially regulated was PI3K-AKT signaling pathway (Fig. 6B). Consistent with these findings, GSEA pathway analysis confirmed that PI3K-AKT signaling pathway and the AKT downstream target FoxO-mediate signaling were substantially reduced in *Tnfaip8*-deficient (indicated as KO) DN cells (Fig. 6C). We found that mTORC1 (by measuring the phosphorylation of S6) as well as mTORC2 (by measuring the phosphorylation of AKT at Ser473) exhibited relatively high levels of phosphorylation in DN cells (Fig. S5A–D). Moreover, DN cells also showed relatively high level of PI3K activity by measuring the phosphorylation of AKT at Thr308 (Fig. S5E–H). These data suggest that the activities of PI3K-AKT/mTOR may be required to maintain for the highly proliferative stages of αβ-lineage development.

Next, we determined whether TNFAIP8 deficiency affected the activities of PI3K-AKT/mTOR. FACS analysis revealed decreased phosphorylation of ribosomal protein S6 in *Tnfaip8*-deficient DN cells but not in DP, CD4SP, and CD8SP cells (Fig. 6D, E). Similarly, TNFAIP8 also had a distinct effect on the activities of PI3K (p-AKT^T308^) and mTORC2 (p-AKT^S473^) (Fig. 6F, G; Fig. S5I, J), suggesting that TNFAIP8 deficiency impedes the activation of PI3K-AKT/mTOR signaling in DN cells. In addition, *Tnfaip8*-deficient DN cells had abnormal PI3K-AKT/mTOR activities, as further indicated by confocal and immunoblot analyses in vitro (Fig. 6H, I). Together, these data suggest that TNFAIP8 is required for the metabolic programming and PI3K-AKT/mTOR activation in DN cells.

### TNFAIP8 promotes PIP3 accumulation and downstream signaling in DN thymocytes

The generation of PIP3, in which PIP2 is phosphorylated locally by PI3K, is a crucial step for recruiting upstream kinases to fully activate both mTORC1 and mTORC2 [30, 31], which prompted us to investigate if TNFAIP8 had a role in the regulation of PIP2/PIP3 metabolism. To test this possibility, we used PIP2/PIP3-specific monoclonal antibodies (mAbs) and found that DN and CD4SP cells exhibited higher PIP2 levels relative to DP cells, with the highest PIP2 levels in CD8SP cells (Figu. S6A, B). Importantly, DN cells displayed the highest PIP3 levels (Fig. S6C, D). Among the different DN subsets, PIP3 levels were remarkably elevated as DN thymocytes progressed through the DN1 stage. The PIP3 level peaked at the DN2 stage and was significantly diminished in cells which had progressed into the DN4 stage (Fig. S6E, F). These data indicate that thymocyte development is accompanied by dynamic regulation both in PIP2 and PIP3 levels.

**Fig. 7:**
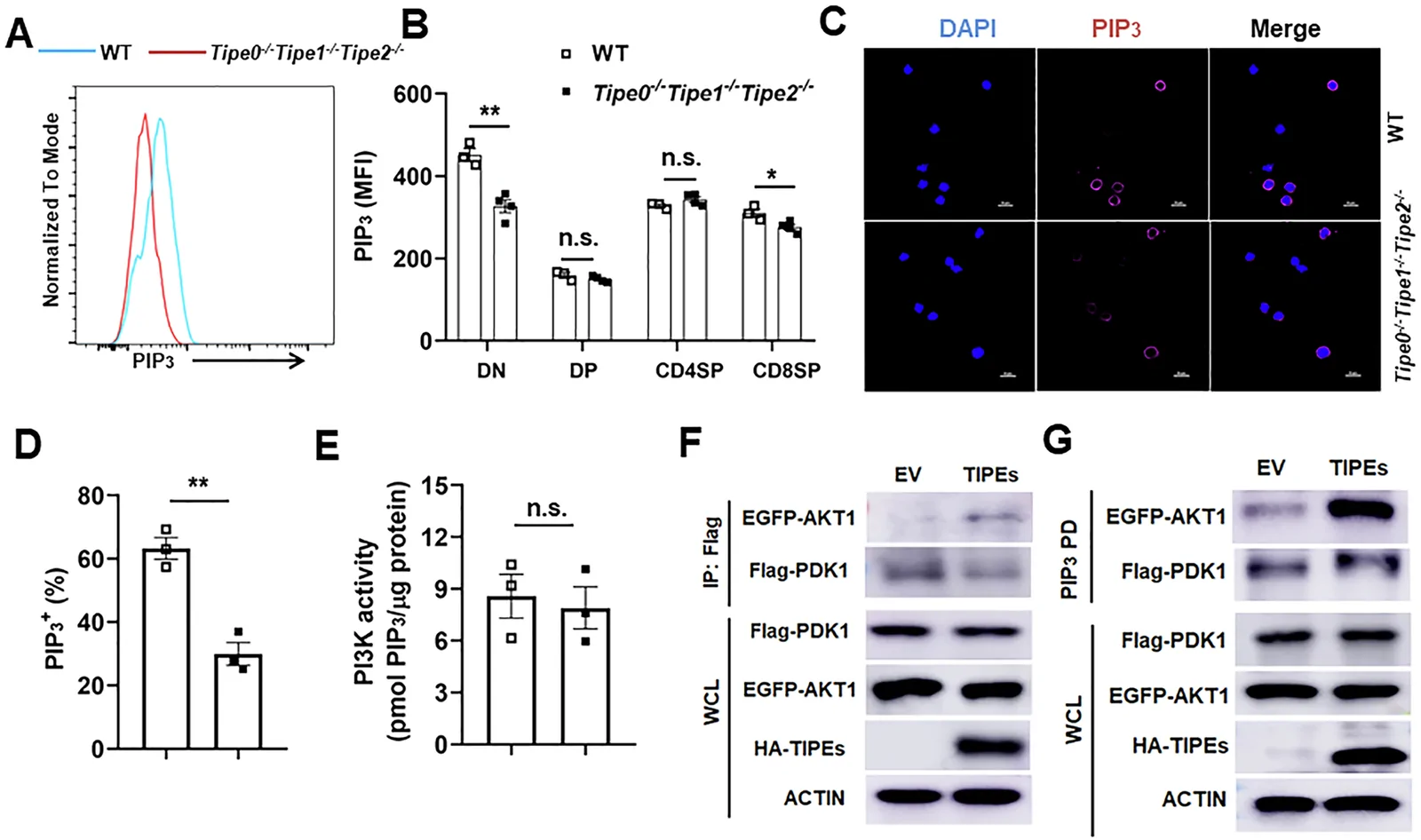
TNFAIP8 affects PIP3 distribution in DN thymocytes and PIP3-mediated downstream signaling. FACS analysis of PIP3 levels in DN, DP, CD4SP, and CD8SP from WT (*n* = 3) and *Tipe0*^*-/-*^*Tipe1*^*-/-*^*Tipe2*^*-/-*^ (*n* = 3) mice, presented as representative plots only for DN (**A**) and summary graph (**B**) for DN, DP, CD4SP, and CD8SP. **C** Confocal microscopy of PIP3, as well as the DNA-binding dye DAPI (colors of labels above images indicate color of molecule (or dye) in image), in WT and *Tipe0*^*-/-*^*Tipe1*^*-/-*^*Tipe2*^*-/-*^ DN thymocytes. Scale bars, 100 μm; original magnification ×4. **D** Quantification of PIP3–positive cells as described in C. Data were pooled from three experiments with *n* ≥ 30 cells per group. **E** PI3K activity in p85 IPs from WT (*n* = 3) and *Tipe0*^*-/-*^*Tipe1*^*-/-*^*Tipe2*^*-/-*^ (*n* = 3) DN cells. **F** Western blot analysis of WCL and co-IP products derived from HEK293T cells transfected with Flag-PDK1, EGFP-AKT1, and the indicated HA-tagged TNFAIP8 family members (HA-TIPEs), including HA-TIPE0, HA-TIPE1, and HA-TIPE2. Where indicated, empty vector (EV) serves as a negative control. **G** Western blot analysis of WCL and PIP3 pull-down products derived from HEK293T cells transfected with the indicated constructs. Where indicated, empty vector (EV) serves as a negative control. Data are representative of three independent experiments. Data are presented as mean ± SEM. Student’s *t* test (**B**, **D** and **E**), *P < 0.05, **P < 0.01, n.s. non-significant.

We next determined the effects of TNFAIP8 on PIP3 generation and PI3K activity. *Tnfaip8*-deficient thymocytes had similar basal PIP2 levels relative to those of WT controls (Fig. S6G). Intriguingly, PIP3 levels, necessary for mTOR activation, were significantly lower in *Tnfaip8*-deficient DN cells (Fig. 7A, B). Confocal microscopic imaging analysis further revealed decreased distribution of PIP3 in *Tnfaip8*-deficient DN cells (Fig. 7C, D). On the contrary, PI3K activity was comparable in these two groups (Fig. 7E). We also observed that TNFAIP8 could dramatically increase the interaction of PDK1 with AKT (Fig. 7F). Furthermore, PIP3 pull-down assay showed that TNFAIP8 dramatically enhanced the interaction of AKT with PIP3 (Fig. 7G). These data indicate that TNFAIP8 could increase the accumulation of PIP3, resulting in enhanced interaction of PDK1 with AKT. Finally, we examined the effect of TNFAIP8 on the activity of PLCγ1, which hydrolyzes the membrane lipid PIP2 to generate diacylglycerol (DAG) and inositol 1,4,5-trisphosphate (IP3) [32]. FACS and immunoblot analyses revealed that TNFAIP8 played a dispensable role in the regulation of PLCγ1 phosphorylation in thymocytes (Fig. S6H–J). Together, these results suggest that TNFAIP8 preferentially regulates PIP2/PIP3 rather than PIP2/DAG/IP3 signaling pathway.

### Notch-driven proliferation and differentiation of thymocytes depend on TNFAIP8

**Fig. 8:**
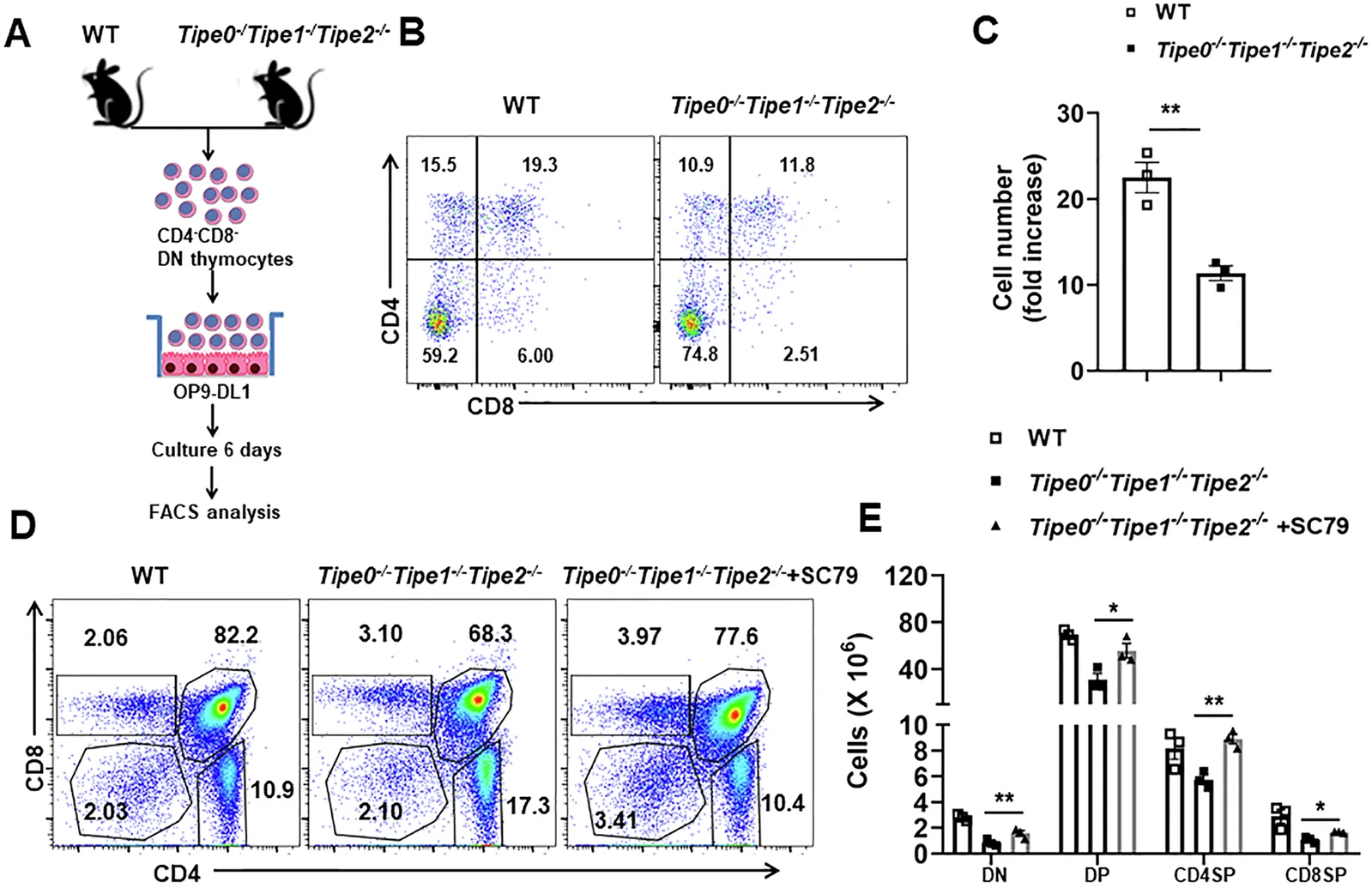
Notch-driven proliferation and differentiation of thymocytes require TNFAIP8. **A** Flowchart of the assay system in vitro. Purified CD4^-^CD8^-^ DN thymocytes from WT (*n* = 3) and *Tipe0*^*-/-*^*Tipe1*^*-/-*^*Tipe2*^*-/-*^ (*n* = 3) mice were cultured on isogenic stromal cells bearing Notch ligand (OP9-DL1) in the presence of IL-7. **B** FACS analysis of the effect of TNFAIP8 proteins on thymocyte differentiation. Shown is a CD4 and CD8 staining of DN cells cultured with OP9-DL1 monolayer for 6 days. **C** DN thymocyte proliferation after TNFAIP8 deletion. Shown are the means for thymocyte expansion in the culture (fold increase=cells recovered/input DN cells). **D**, **E** FACS analysis of thymic subpopulations based on surface staining of CD4 and CD8 from WT (*n* = 4), *Tipe0*^*-/-*^*Tipe1*^*-/-*^*Tipe2*^*-/-*^ (*n* = 3), and *Tipe0*^*-/-*^*Tipe1*^*-/-*^*Tipe2*^*-/-*^ (*n* = 3) mice injected with SC79, presented as a representative FACS plot (**D**) and summary graph (**E**). Data are representative of three independent experiments. Data are presented as mean ± SEM. Student’s *t* test (**C** and **E**), *P < 0.05, **P < 0.01.

To investigate a potential role for TNFAIP8 in Notch-driven thymocyte progression, we used an in vitro system with OP9 stromal cells expressing the Notch ligand DL1 (OP9-DL1) (Fig. 8A), as previously described [33]. As in the work connecting mTOR to Notch, these cultures were supplemented with IL-7 [33]. Consistent with previous report, WT DN cells exhibited rapid proliferation (more than 20-fold increase) and differentiation into DP thymocytes (~20-30% of cells). By contrast, *Tnfaip8*-deficient DN cells proliferated and differentiated less efficiently on the OP9-DL1 (Fig. 8B, C). These results demonstrate a requirement for TNFAIP8 in the proliferation and differentiation of pre-T cells in a Notch-dependent setting.

To further determine whether the impaired activation of AKT-mTOR axis contributed to the T cell developmental abnormalities in *Tipe0*^*-/-*^*Tipe1*^*-/-*^*Tipe2*^*-/-*^ mice, we performed a functional rescue experiment by administrating SC79, a selective membrane-permeable AKT activator that binds to the PH domain of AKT, to *Tipe0*^*-/-*^*Tipe1*^*-/-*^*Tipe2*^*-/-*^ mice every other day for a total of 10 times. SC79 treatment partially restored thymic cellularity compared with untreated *Tipe0*^*-/-*^*Tipe1*^*-/-*^*Tipe2*^*-/-*^ mice (Fig. S7A). Further analysis of T cell developmental stages showed that a partial restoration of cell frequencies and cell numbers of DN, DP, CD4SP, and CD8SP cells as compared with control mice (Fig. 8D, E). Additionally, SC79 administration substantially increased both expansion and the efficiency of differentiation of Notch-stimulated *Tipe0*^*-/-*^*Tipe1*^*-/-*^*Tipe2*^*-/-*^ DN thymocytes in the OP9-DL1 co-culture system, attaining almost to WT values (Fig. S7B–D). These data suggest that hypoactivation of AKT is a key mechanism by which loss of TNFAIP8 results in defects during T lymphopoiesis in the thymus. Therefore, these observations suggest that TNFAIP8 facilitates T cell development by fine-tuning cellular metabolism via AKT-mTOR axis.

## Discussion

TNFAIP8 proteins are best known for their roles in driving cell polarization (head and tail formation) and in specifying the directionality of cell migration upon chemokine stimulation. TIPE2, the most well- documented TNFAIP8 family member, is an essential regulator of innate and adaptive immunity that maintains immune homeostasis [17]. TIPE2 regulates the thymus egress of Treg cells and TIPE2 deficiency results in the accumulation of Treg cells in the thymus [34]. Consistent with this report, we observed the percentage of Treg cells was increased, but the absolute number of Treg cells was significantly reduced in *Tipe0*^*-/-*^*Tipe1*^*-/-*^*Tipe2*^*-/-*^ mice due to the overall reduction of thymic cellularity. TNFAIP8 polarity proteins are required for deployment of T cells and TIPE0 and TIPE2 appears to have overlapping functions since deficiency in both yields much stronger phenotype [35]. Howerver, previous studies suggest it plays a more limited role in thymocyte development and differentiation [17, 36]. We report here a crucial role of three TNFAIP8 family members, i.e., TIPE0, TIPE1, and TIPE2 in thymocyte development, which act in a redundant manner. Genetic deletion of all three, but not one or two alone, caused dramatic reductions in thymic size, cellularity, and architecture, as well as in the total numbers of DN, DP, CD4SP, and CD8SP thymocytes. The reduction in DN cell numbers is of importance because there may be insufficient quantities of these cells in *Tipe0*^*-/-*^*Tipe1*^*-/-*^*Tipe2*^*-/-*^ thymuses to generate normal numbers of mature SP cells. The redundancy in TNFAIP8 function during thymic development may ensure that adequate T cells are generated despite mutations in one or two of the *Tipe* genes.

Our data revealed that the functions of TNFAIP8 in T lymphopoieisis are at least in part through promoting proliferation of DN cells, especially DN4 cell, but not apoptosis of thymocytes. Because PI3K-AKT/mTOR pathway is impaired in *Tnfaip8-*deficient thymocytes, we propose that TNFAIP8 promotes T lymphopoiesis at least in part by regulating this signaling axis. Consistent with this interpretation, targeted mutations of both *Akt1* and *Akt2* in the thymus recapitulate the phenotypes of *Tnfaip8* deletion reported in this study, namely reduced T lymphopoiesis, defective DN proliferation, decreased metabolism, and developmental blockade of DN3-DN4 transition [37, 38]. mTORC1 inhibition by rapamycin treatment or the genetic deletion of the *Raptor* gene and mTORC2 inhibition by deleting *Rictor* gene using Vav-Cre or Lck-Cre also partially recapitulate the phenotypes of *Tnfaip8* deletion [9, 10, 13]. Based on an established in vitro model [33, 39], we also observed a remarkable role of TNFAIP8 in Notch-driven thymocyte proliferation and differentiation. Because the *Tnfaip8* defects were partially recused by constitutive AKT activation in vivo and in vitro, we suggest that PI3K-AKT axis is likely the downstream target of TNFAIP8 in our system.

Phosphatidylinositol and its phosphorylated derivatives, the phosphoinositides play a major role as signaling molecules, affecting an impressively diverse set of biological functions [40]. The phosphatidylinositol transfer proteins (PITPs) are key regulators of phosphoinositide signaling. Like PITPs, the mammalian TNFAIP8 exhibits phospholipid-transfer activity, and are proposed to serve as nanoreactors which present phosphoinositides to the appropriate enzymes for modification. By “presenting” PIP2 to PI3K and enhancing the generation of PIP3 at PIP3-containing membranes, TIPE2 reinforces a positive feedback loop that amplifies the PI3K signaling [20]. Similarly, TIPE0 is able to extract PIP2 from the membrane, but only in the presence of PIP3, indicating that this effect occurs when a cell is undergoing some form of PI3K-activating stimulation [21]. The ability of TNFAIP8 to enhance PI3K activity indicates that, rather than functioning as a bona fide transfer protein, TNFAIP8 acts as a presentation module in a manner similar to that of PITPs. This is a previously undescribed mechanism regulating PI3K-dependent signaling, such as the AKT pathway. The results reported here provide evidences that the combined activities of TIPE0, TIPE1, and TIPE2 are crucial for thymocyte development, although it appears that one of them is sufficient to maintain T cell production. This potential redundancy in function may ensure that adequate levels of PIP3 are maintained to drive cell proliferation.

PIP3 generation on the plasma membrane is a critical step for recruiting PDK1/AKT or other upstream kinase such as mTORC2 to fully activate AKT kinases. Conceivably, the abnormal accumulation/distribution of PIP3 will potently affect AKT kinase activity [41]. Intriguingly, we found DN cells showed higher PIP3-AKT/mTOR activity compared with DP, CD4SP, and CD8SP cells. This characteristic of DN cells might be conducive for its rapid proliferative potential, which is important for maintaining normal thymic cellularity. Although TNFAIP8 played a dispensable role in regulating basal PIP2 levels in DN cells, its deficiency resulted in significantly reduced PIP3 accumulation in DN cells. Consistent with this result, we also found that *Tnfaip8-*deficient DN cells exhibited lower amounts of phosphorylated AKT-T308 and AKT-S473, suggesting that TNFAIP8 might affect the generation and/or removal of PIP3 to strengthen PI3K-mediated downstream signaling. However, we observed that both PI3K activities were comparable in WT and *Tnfaip8*-deficient DN cells. Therefore, we propose that TNFAIP8 might regulate PIP3 accumulation through affecting the ability of PIP2 “presentation” rather than the activities of PI3K (kinase).

In summary, this work provides pivotal insights into the molecular mechanisms governing T lymphopoiesis and maintenance of the peripheral T lymphocyte pool. It reveals previously unknown roles of TNFAIP8 proteins in the control of regulatory programs for thymocyte development. Thus, targeting the TNFAIP8 signaling pathway may be useful for controlling T cell-mediated immunity.

## Materials and methods

### Mice

C57BL/6J (B6) wild-type (WT) mice were originally purchased from Shanghai Biomodel Organism Science & Technology Development Company Limited (Shanghai, China). *Tipe1*^*-/-*^ B6 mice were generated with CRISPR/Cas9-mediated genome editing by Shanghai Biomodel Organism Science & Technology Development Company Limited [23]. *Tipe0*^*-/-*^ B6 mice and *Tipe2*^*-/-*^ B6 mice were prepared as described [36, 42]. Single-gene homozygous KO mice were used to generate double and triple-knockout combinations. All mice were apparently healthy and bred normally in homozygous form, except for the female *Tipe0*^*-/-*^*Tipe1*^*-/-*^*Tipe2*^*-/-*^ mice, which often exhibited unsuccessful breastfeeding. Therefore, *Tipe0*^*-/-*^*Tipe1*^*-/-*^*Tipe2*^*-/-*^ mice were maintained by crossing male *Tipe0*^*-/-*^*Tipe1*^*-/-*^*Tipe2*^*-/-*^ and female *Tipe0*^*+/-*^*Tipe1*^*-/-*^*Tipe2*^*-/-*^ mice. Genotyping was performed by PCR using specific primers for each gene. CD45.1^+^ B6 mice were obtained from Yinming Liang (Xinxiang Medical University). CD45.1^+^CD45.2^+^ mice were generated by mating CD45.1^+^ B6 WT mice with CD45.2^+^ B6 WT mice. Both males and females at 6-12 weeks of age were used unless otherwise noted. Group allocation for all experiments was randomized and not blinded. Sample analyses were not blinded. All mice were housed in the Xinxiang Medical University Animal Care Facilities under pathogen-free conditions. All animal procedures were preapproved by the Institutional Animal Care and Use Committee of Henan Medical University.

### Competitive bone marrow chimeras

Bone marrow chimeric mice were generated by radiation as previously described [36, 42]. Briefly, bone marrow (BM) cells were collected from the tibias and femurs of age-matched WT (CD45.1^+^) or *Tipe0*^*-/-*^ *Tipe1*^*-/-*^*Tipe2*^*-/-*^ mice (CD45.2^+^). BM cells were then mixed at a 1:1 ratio (10 million cells in total) and transferred intravenously (i.v.) into WT (CD45.1^+^CD45.2^+^) recipient mice lethally irradiated at 900 rads in two doses (450 rads each) 3 h apart, followed by reconstitution for eight weeks.

### Flow cytometry

Single-cell suspensions were isolated and prepared from thymus, spleen, LNs, and PBCs as described [43]. The cells were stained with the indicated fluorescence-conjugated antibodies after anti-mouse CD16/CD32 antibody blockade. Dead cells were stained with a Live/Dead fixable aqua dead cell stain kit (Invitrogen) and were gated out in the analysis. Antibodies used in the current study are listed in Supplementary Table S1. For analysis of surface markers, cells were stained in PBS containing 2% FBS with appropriate surface antibodies. Foxp3 and ki67 staining was performed as per the manufacturer’s instruction (eBioscience). For detection of phosphorylated signaling proteins, after staining for surface antigens, cells were fixed with Phosflow Lyse/Fix buffer (BD Bioscience), followed by permeabilization with Phosflow Perm buffer I (BD Bioscience), and staining with antibodies to phosphor-AKT (Ser 473), phosphor-S6 (Ser235/236), phosphor-AKT (Thr 308), or phosphor-PLC-γ (Tyr 783). Fluorescence-activated cell sorting (FACS) data were acquired on FACSCanto (BD Bioscience) or Attune NxT (Invitrogen) instruments and analyzed with FlowJo software (v10.0; TreeStar).

### Proliferation and apoptosis analyses

Mice were inoculated with 200 μl of a 10 mg/ml BrdU stock intraperitoneally 4 h before the thymus was harvested. Thymocytes were surface-stained, and then intracellular staining of BrdU was performed using an APC BrdU Flow kit as per the manufacturer’s instruction (BD Pharmingen). For thymocyte apoptosis assay, cells were stained with surface FACS antibodies, and then stained with FITC Annexin V following the manufacturer’s instruction (KeyGen Biotechnology).

### DN purification and culture

Thymocytes were isolated and CD4^-^CD8^-^ DN subsets were purified from pooled thymocytes by CD8 depletion using biotinylated anti-CD8 and IMag streptavidin particles followed by CD4 depletion using anti-CD4 microbeads and magnetic activated cell sorting (BD Bioscience). In the purified cells, more than 95% were CD4^-^ and CD8^-^. For in vitro differentiation of DN thymocytes, DN cells were plated on stromal cell monolayers (OP9-DL1, provided by Zhuan Li, Southern Medical University) in the presence of 5 ng/ml IL-7 (PeproTech) for 6 days. They were maintained in complete medium containing 15% FBS and supplemented as described previously [44]. Harvested cultures were passed through 40-μm filters to remove the stromal cells, counted, and determined by FACS.

### PIP2 and PIP3 assays

For PIP2 and PIP3 measurements by immunostaining, biotinylated monoclonal anti-PIP2 antibody (clone 2C11, Echelon Bioscience) and anti-PIP3 antibody (clone RC6F8, Echelon Bioscience) were used as previously described [28]. In brief, thymocytes were stained for surface markers and immediately fixed with 4% paraformaldehyde (PFA) and 0.2% glutaraldehyde (GA, Macklin) at room temperature for 15 min and on ice for 30 min. Cells were then blocked and permeabilized for 45 min on ice with PIPES buffer (pH 7.0, Macklin) containing 5% (v/v) normal goat serum (NGS, Invitrogen), 50 mM NH4Cl and 0.5% saponin (MedChemExpress). Anti-PIP2 or anti-PIP3 antibody (2.0 μg/ml) was applied in PIPES buffer with 5% NGS and 0.1% saponin for 1 h. Cells were washed twice with PIPES buffer and resuspended in PIPES buffer with fluorescent conjugated streptavidin for 45 min. After three washes in PIPES buffer, cells were resuspended in PBS containing 2% FBS and analyzed by flow cytometry.

### Metabolic measurement

Purified DN thymocytes were seeded at a density of 5 × 10^5^ per well in a 24-well Seahorse assay plate. The oxygen consumption rate (OCR) measurement was conducted with the Seahorse XFe24 Extracellular Flux Analyzer (Aglient Technologies). OCR (pmol/min) was determined as indicated under different experimental conditions using the XF Cell Mito stress test kit upon cell treatment with oligomycin (1 µM), the mitochondrial uncoupler carbonyl cyanide-4-(trifluoromethoxy) phenylhydrazone (FCCP) (2 µM), and rotenone/antimycin A (0.5 µM) as per the manufacturer’s instructions (Aglient Technologies).

### RNA sequencing (RNA-seq) analysis

DN thymocytes were purified from WT and *Tipe0*^*-/-*^ *Tipe1*^*-/-*^*Tipe2*^*-/-*^ mice. Total RNA of DN cells was then extracted using the mirVana miRNA Isolation Kit (Ambion). RNA integrity was evaluated using the Agilent 2,100 Bioanalyzer (Agilent Technologies). The samples with RNA Integrity Number (RIN) ≥ 7 were subjected to sequence by the Solexa high-throughput sequencing service (OEBiotech Co., Ltd.). Differentially expressed genes were identified using the DESeq (2012) functions estimateSizeFactors and nbinomTest. Log-fold changes of upregulated or downregulated mRNAs between the two groups were selected with a significance threshold of *P* < 0.05. Only genes with more than twofold changes and *P* value of less than 0.05 were considered for further analyses. Kyoto Encyclopedia of Genes and Genomes (KEGG) pathway enrichment analysis based on differently expressed genes (DEGs) was performed to investigate gene groups that participate in common biological responses or possess related functions. Pathway analysis was also performed using gene set enrichment analysis (GSEA) (www.broadinstitute.org/gsea/downloads.jsp) with default settings from GSEA database. GSEA does not focus on only significantly/highly changed genes but examines all the genes that belong to a certain biological process instead. RNA-seq data were deposited in the National Center for Biotechnology Information BioProject under accession number PRJNA1335577. The sequence read archive records will be accessible with the following link after the indicated release date (https://www.ncbi.nlm.nih.gov/sra/PRJNA1335577).

### Plasmid constructs

TIPE2-HA expression plasmid was described previously [45]. HA-tagged TIPE0 and TIPE1 plasmids were constructed using PCR-generated fragments, which were cloned into pcDNA3.0 eukaryotic expression vector (Invitrogen) with an HA tag at the C terminus. Flag-PDK1 and EGFP-AKT1 plasmids were purchased from Miaoling Plasmid Platform. All constructs were confirmed by DNA sequencing.

### Immunoprecipitation (IP), PIP3 pull-down and immunoblotting analyses

Cells were collected, washed with cold PBS, and lysed on ice for 30 min in RIPA buffer (Beyotime Biotechnology) supplemented with fresh protease inhibitors (Complete Ultra Tablets Mini easypack, Roche) and phosphatase inhibitor cocktail tablets (phos STOP easypack, Roche). The protein concentrations of whole cell lysates (WCL) were measured using protein assay reagent (Beyotime Biotechnology). The same amounts of WCL were resolved by SDS-PAGE and immunoblotted with indicated antibodies. For IP analysis, WCL were incubated with the distinct agarose beads for Flag (Santa Cruz Biotechnology) or PI(3,4,5)P3 (Echelon Bioscience). The recovered immune-complex products were washed three times with IP buffer before being resolved by SDS-PAGE and immunoblotted with the indicated antibodies.

### Confocal microscopy

Purified DN thymocytes were isolated and fixed with 4% PFA in PBS for 15 min, then permeabilized in PBS containing 0.1% Triton X-100 for 10 min and blocked with PBS containing 3% BSA for 1 h. Cells were first incubated overnight at 4 °C with antibody to AKT phosphorylated at Thr308 (Cell Signaling Technology) or antibody to PtdIns(3,4,5)P3 (Echelon Bioscience) in 3% BSA, then were incubated for 1 h with donkey anti-rabbit IgG (H + L) highly cross-adsorbed secondary antibody Alexa Fluor 488 (Invitrogen) or F(ab’)2-goat anti-mouse IgG (H + L) secondary antibody APC in 3% BSA. Sides were dried and covered with ProLong Gold with 4, 6-diamidino-2-phenylindole (DAPI, Invitrogen). Images were acquired on a Nikon A1R microscopy.

### Administration of AKT activator to mice

For in vivo activation of AKT in mice, SC79 (75 μg/kg, MedChemExpress) dissolved in Dimethylsulfoxide (DMSO) was administrated to *Tipe0*^*-/-*^ *Tipe1*^*-/-*^*Tipe2*^*-/-*^ mice by intraperitoneal injection every other day starting on day 1. Mice were euthanized for experiments on day 22 (10 times in total).

### Statistical analysis

All quantitative data are presented as means ± SEM of two or three experiments. All statistical analyses of experiments were performed using two-tailed Student *t* test, or One-way ANOVA with multiple comparisons. *P* < 0.05 was considered statistically significant. Data analysis and representation were performed with the Prism 8.0 for Windows (Graphpad Software).

## Supplementary information

Original qPCR

Original Western blots

Supplementary Information

## Data Availability

All data needed to evaluate the conclusions of this paper are already present in the article file and the supplemental information. Additional related data needs to be requested from the corresponding author.
