# Supplementary material for: TNFAIP8 polarity proteins mediate T cell development by propagating lipid second messenger signaling in thymocytes
Source: Cell Death Dis. 2026 Jul 27;17(1):897. doi: 10.1038/s41419-026-09100-6 (PMC13686962; doi:10.1038/s41419-026-09100-6)
Supplement: Supplementary file 1 [file 41419_2026_9100_MOESM1_ESM.pdf]

Original qPCR

Figure S2D

Tipe0			*Tipe1*			*Tipe2*		
0.008	0.007	0.012	0.0045	0.0014	0.0047	0.044	0.067	0.076
