# Supplementary material for: TNFAIP8 polarity proteins mediate T cell development by propagating lipid second messenger signaling in thymocytes
Source: Cell Death Dis. 2026 Jul 27;17(1):897. doi: 10.1038/s41419-026-09100-6 (PMC13686962; doi:10.1038/s41419-026-09100-6)

Original western blots

Figure 6I

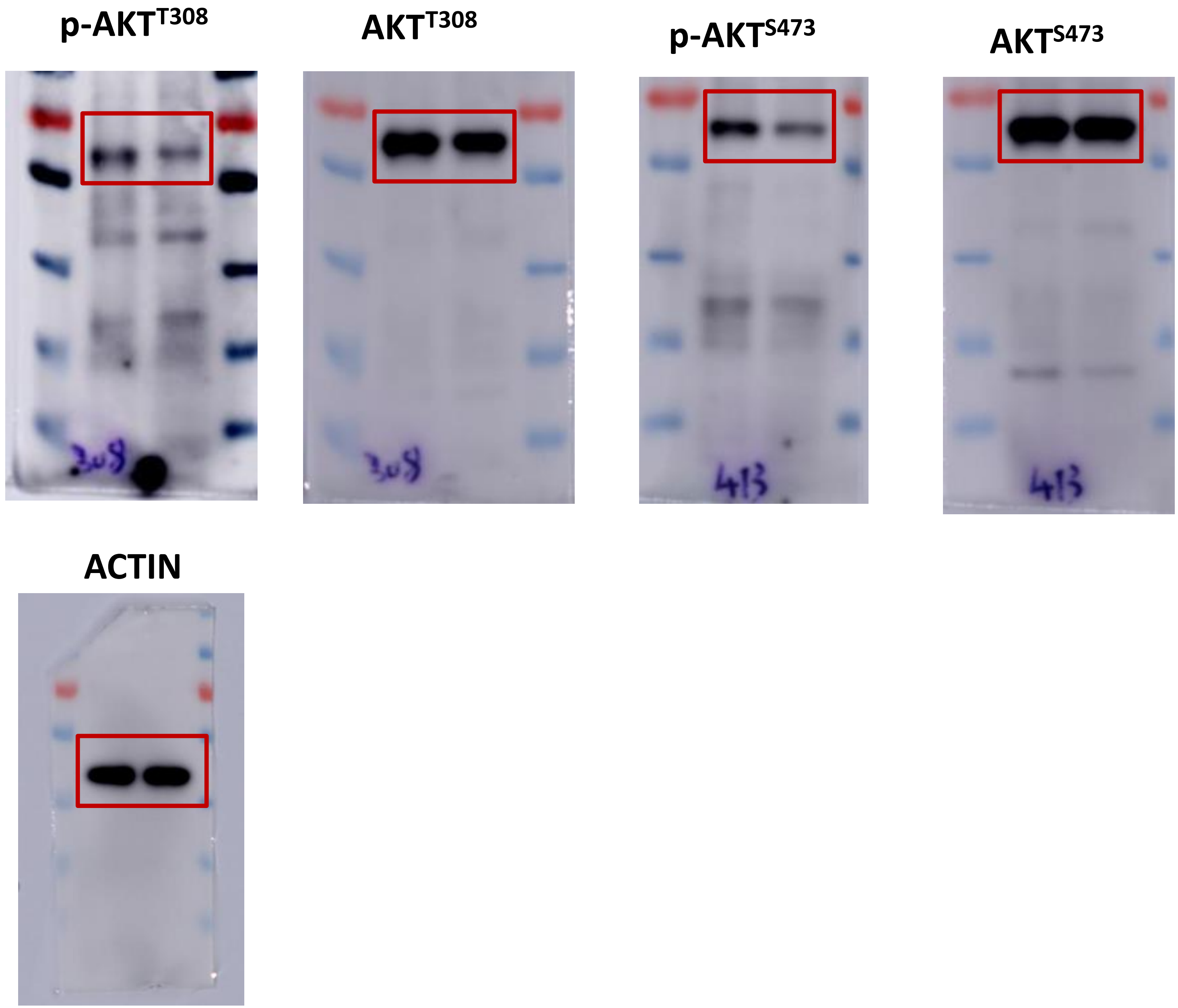

Figure 7F

IP: EGFP-AKT1

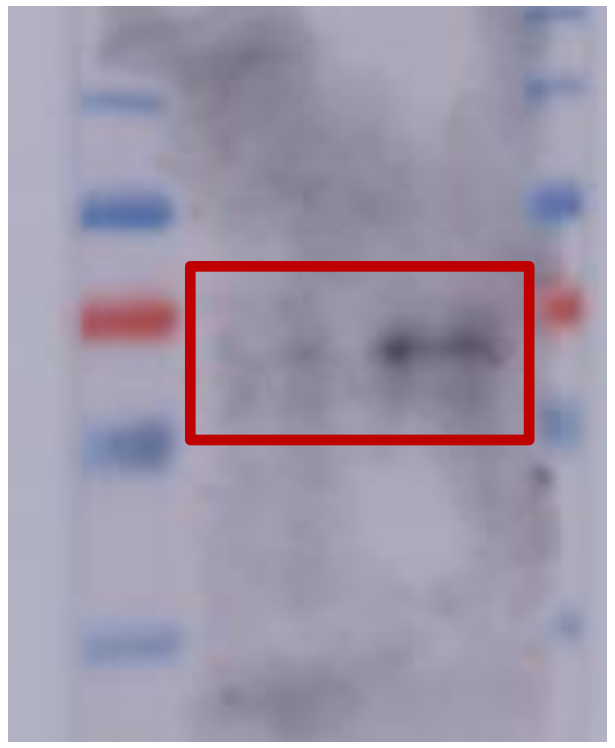

IP: Flag-PDK1

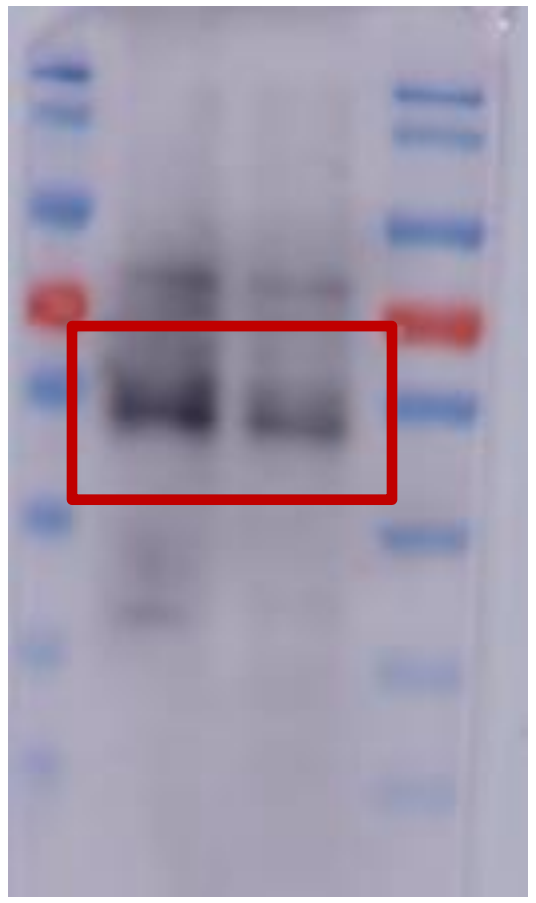

WCL: Flag-PDK1

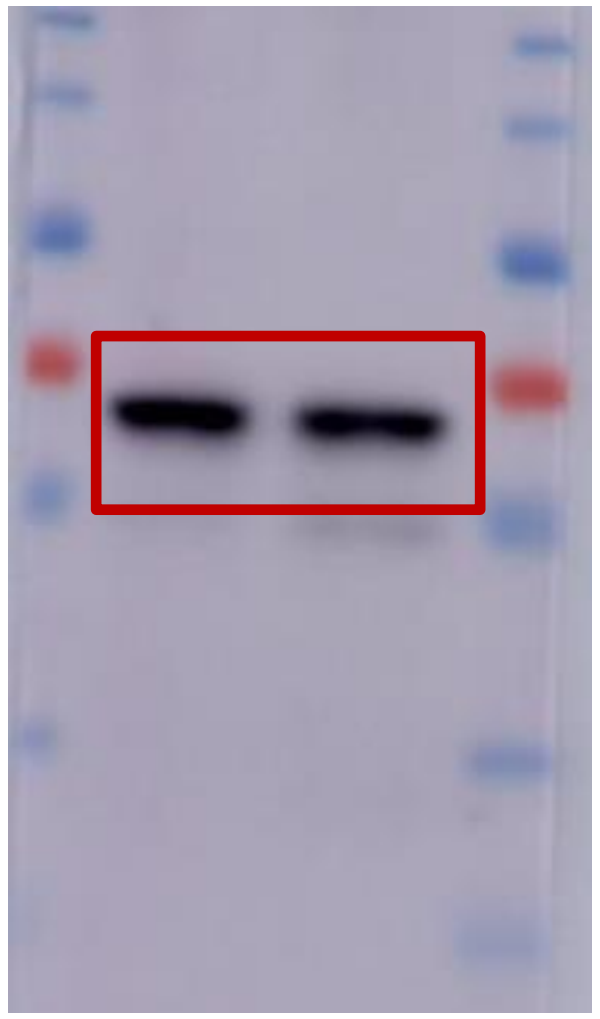

WCL: EGFP-AKT1

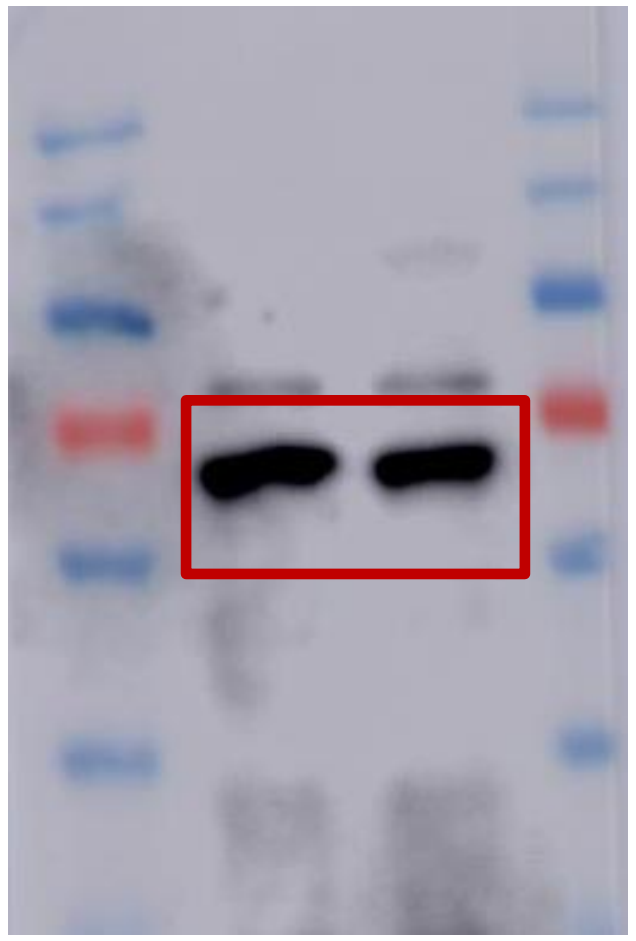

WCL: HA-TIPEs

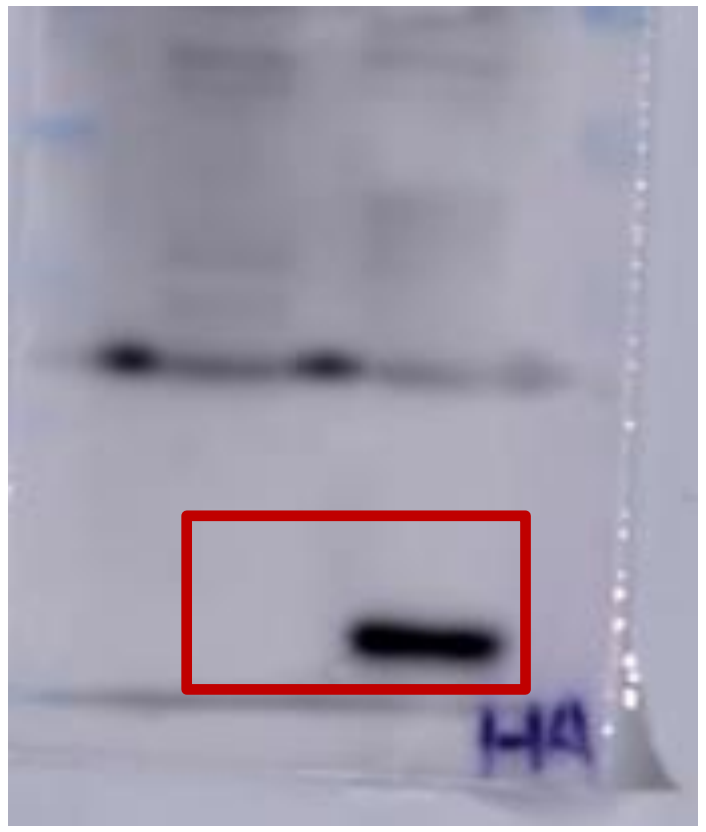

WCL: ACTIN

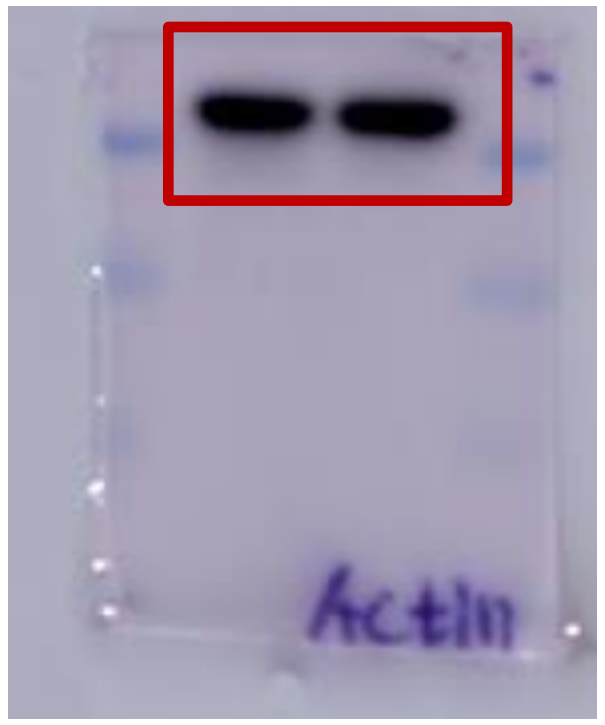

Figure 7G

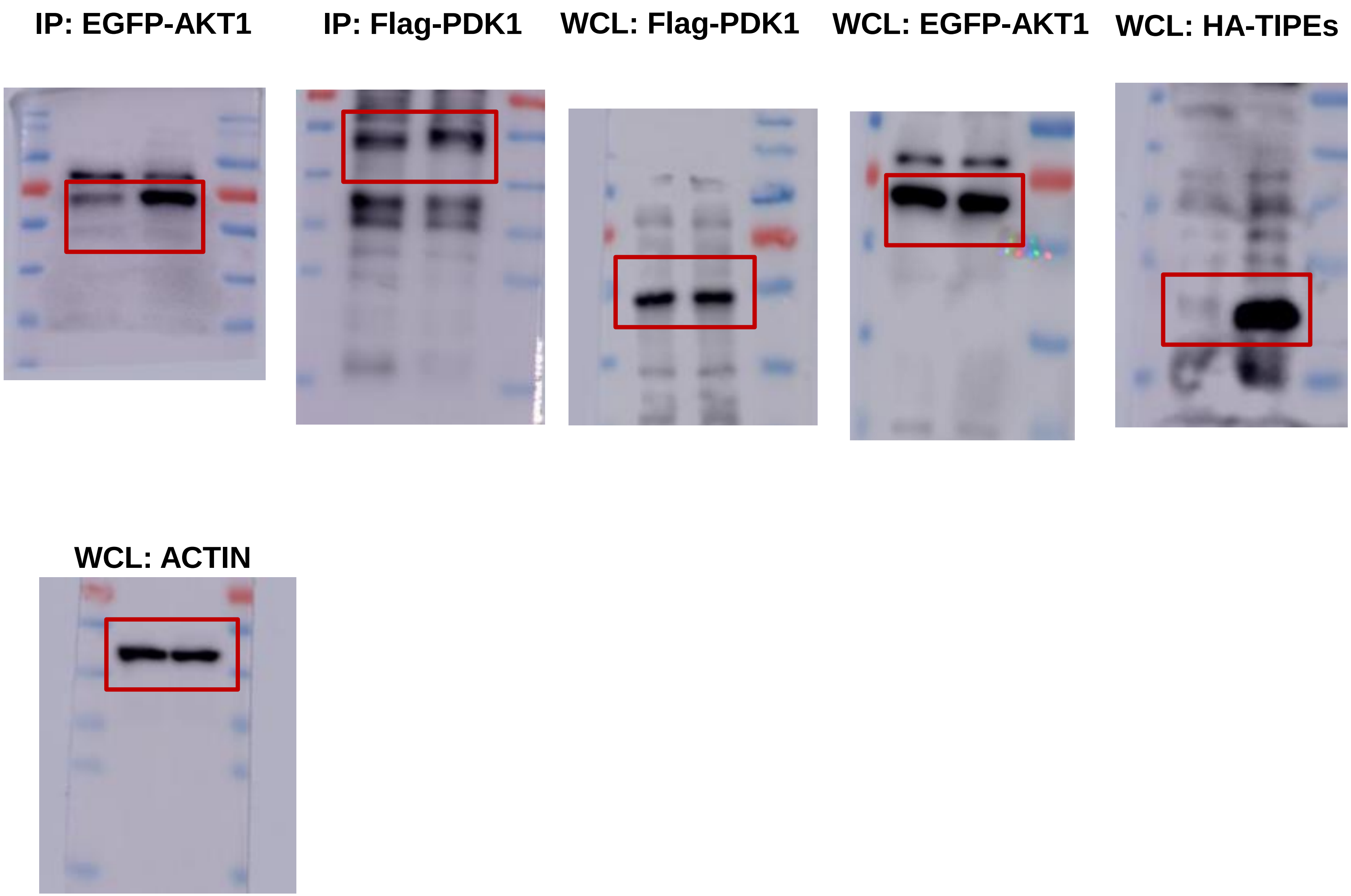
IP: EGFP-AKT1
IP: Flag-PDK1
WCL: Flag-PDK1
WCL: EGFP-AKT1
WCL: HA-TIPEs
WCL: ACTIN

Figure S6J

p-PLC-γ

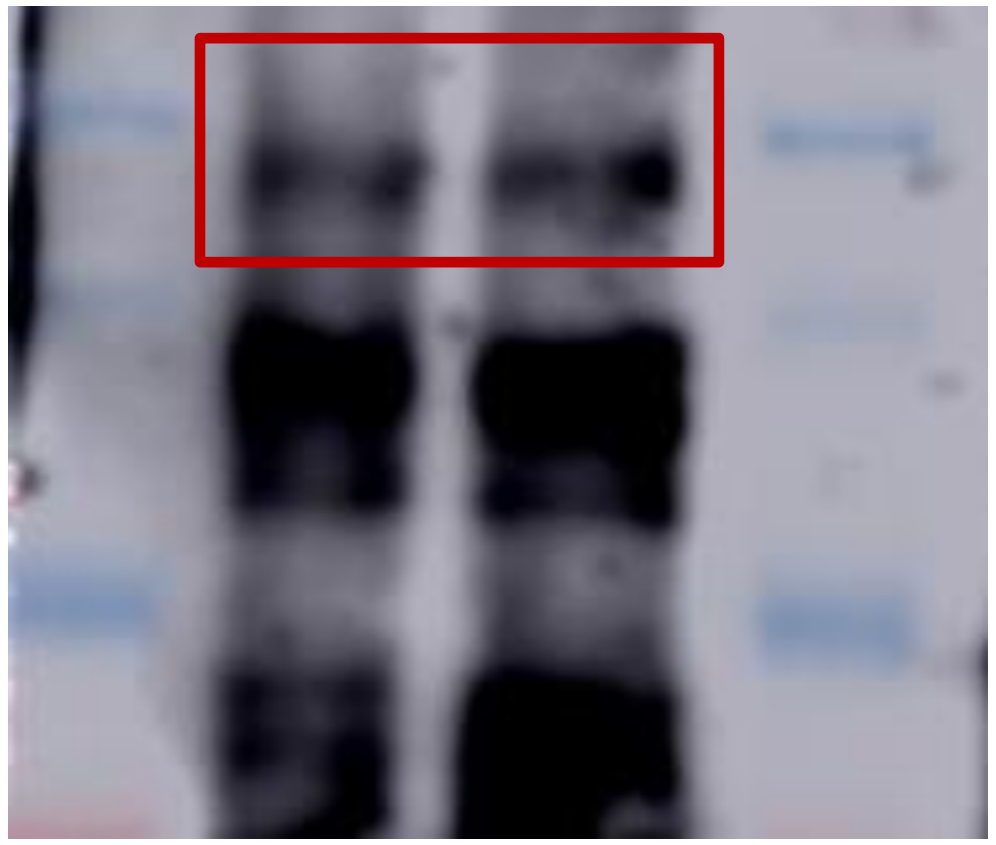

PLC-γ

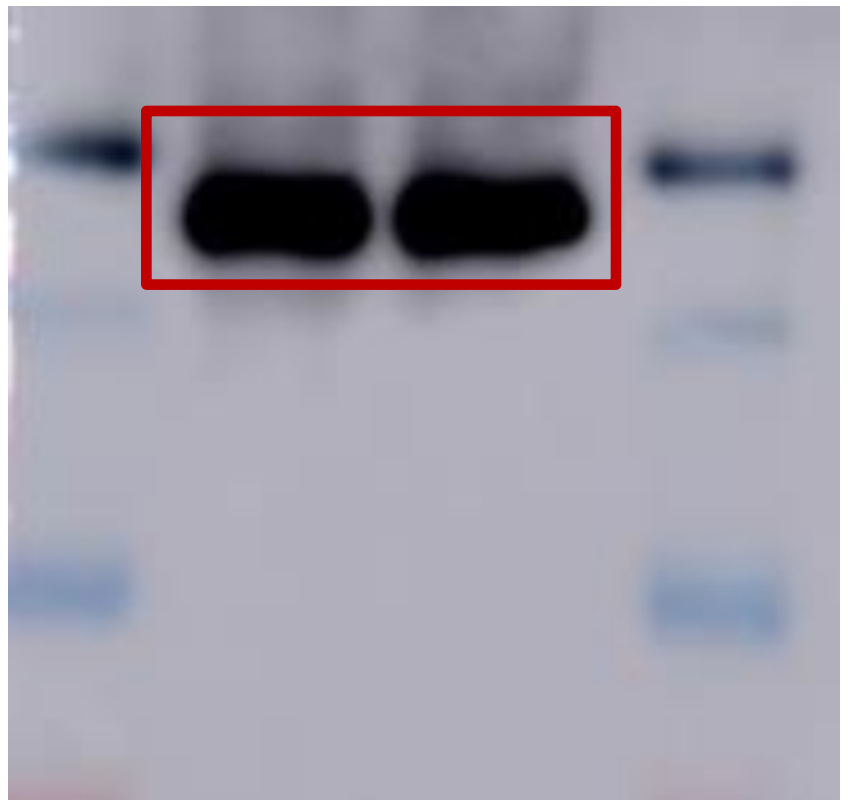

Supplement: Supplementary file 2 [file 41419_2026_9100_MOESM2_ESM.pdf]
