# Supplementary material for: TNFAIP8 polarity proteins mediate T cell development by propagating lipid second messenger signaling in thymocytes
Source: Cell Death Dis. 2026 Jul 27;17(1):897. doi: 10.1038/s41419-026-09100-6 (PMC13686962; doi:10.1038/s41419-026-09100-6)
Supplement: Supplementary file 3 [file 41419_2026_9100_MOESM3_ESM.pdf]

Supplemental information

TNFAIP8 polarity proteins mediate T cell development by propagating lipid second messenger signaling in thymocytes

Jingtao Gao, Cun Guo, Yang Lv, Han Wang, Sheng Chen, Peili Zhuang, Miaomiao Song, Xueqin Tian, Shan Jiang, Wei Lu, Lu Zhang, Peiqing Zhao, Xiaohan Wu, Xiangfeng Song, Xiaohong Kang, Youhai H. Chen, Tingmin Chang, Hui Wang, and Yunwei Lou.

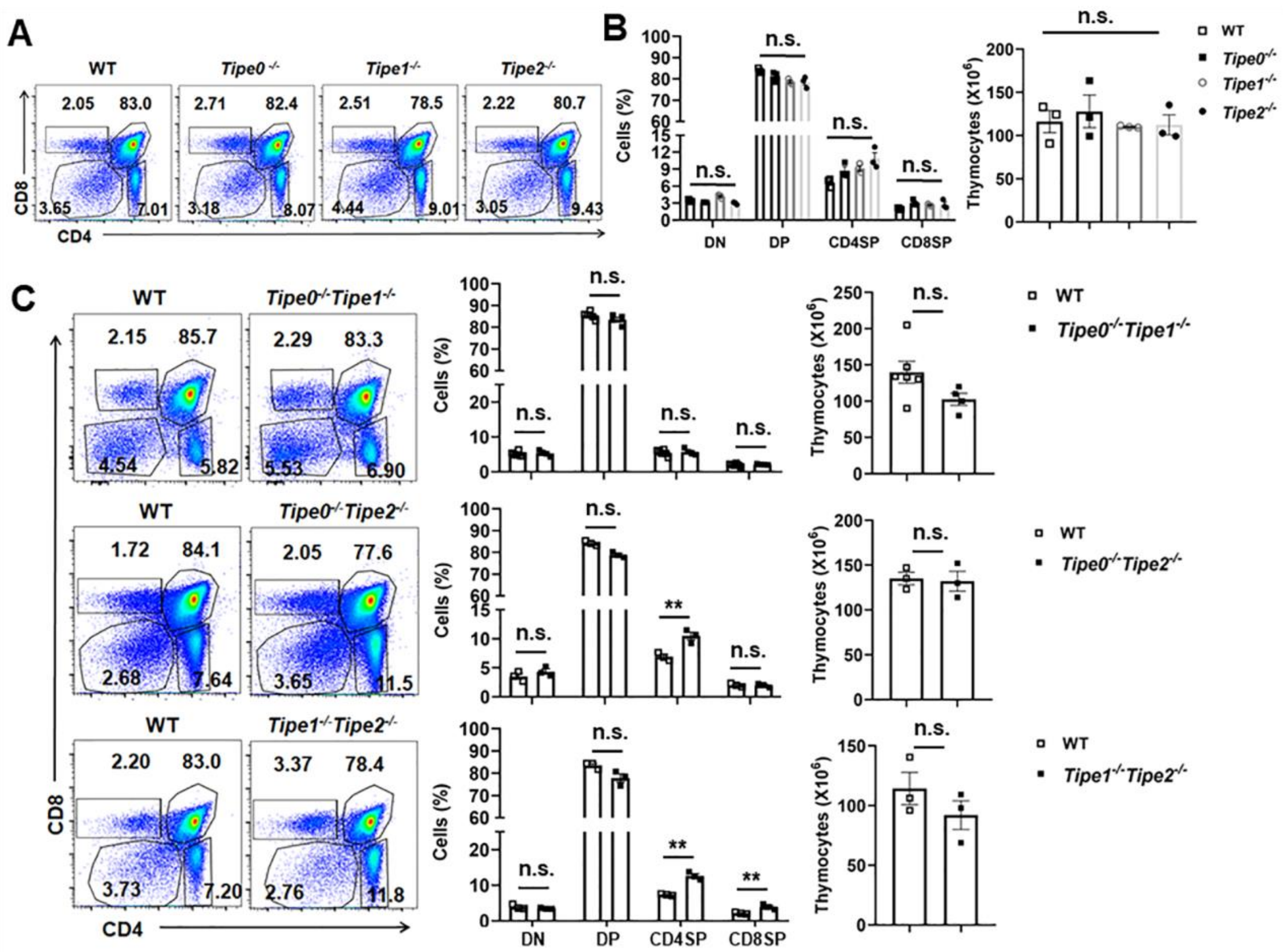

Figure S1. Effect of single *Tipe* gene knockout and double *Tipe* gene knockout on T cell development.

(A-B) FACS analysis of thymic subpopulations based on surface staining of CD4 and CD8 from WT (n=3), *Tipe0*$^{-/-}$ (n=3), *Tipe1*$^{-/-}$ (n=3) and *Tipe2*$^{-/-}$ (n=3) mice, presented as a representative FACS plot (A) and summary graphs (B). (C) FACS analysis of thymic subpopulations based on surface staining of CD4 and CD8 from WT (n=3-6), *Tipe0*$^{-/-}$*Tipe1*$^{-/-}$ (n=4), *Tipe0*$^{-/-}$*Tipe2*$^{-/-}$ (n=3) and *Tipe1*$^{-/-}$*Tipe2*$^{-/-}$ (n=3) mice, presented as representative FACS plots (left panel) and summary graphs (right panel). Data are representative of at least three independent experiments. Data are presented as mean ± SEM. Student's *t* test (B and C), $^{**}P<0.01$, n.s. non-significant.

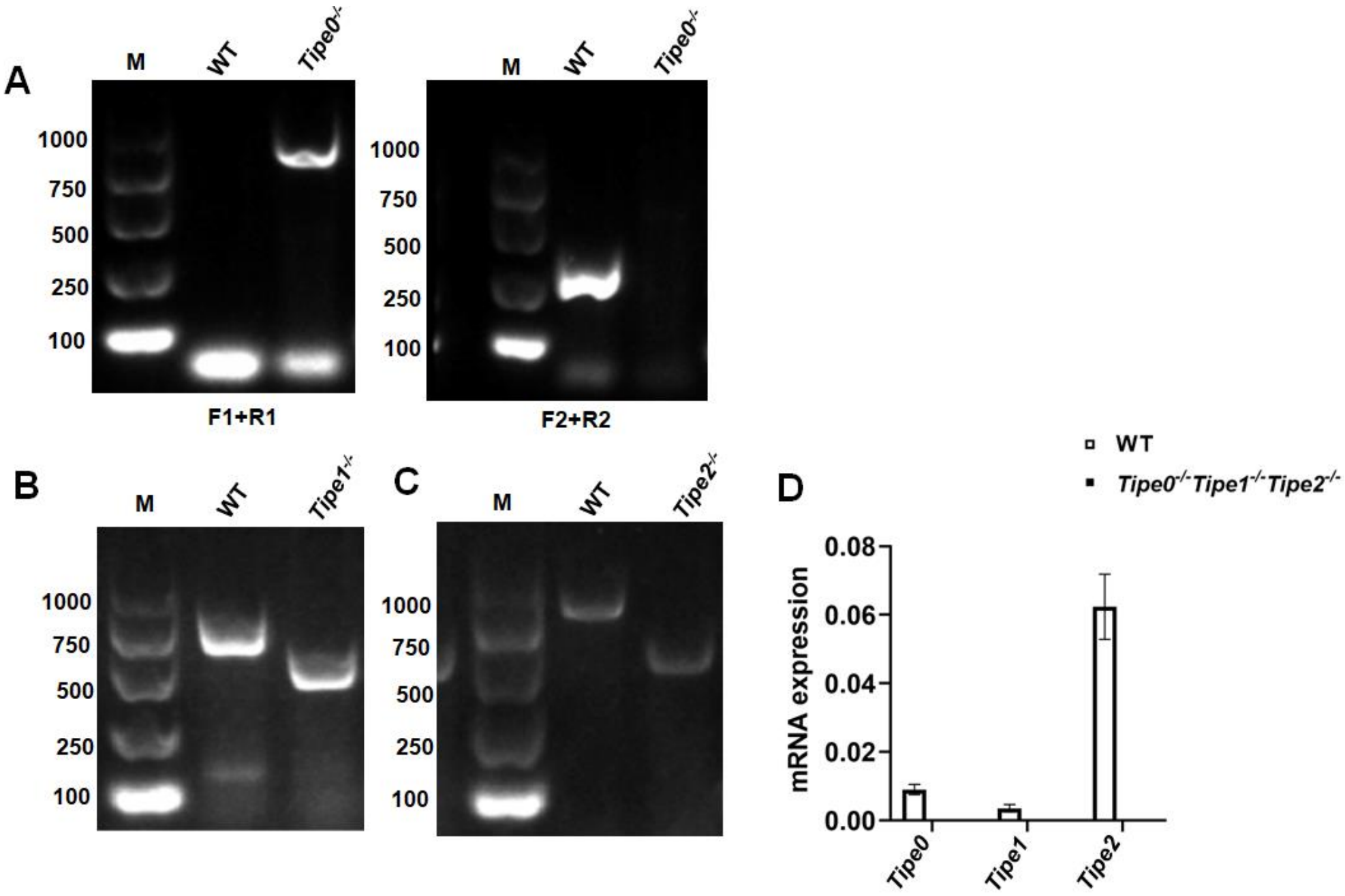

Figure S2. Generation and confirmation of *Tipe0*$^{-/-}$*Tipe1*$^{-/-}$*Tipe2*$^{-/-}$ mice.

(A) Genotyping PCR on mice tail genomic DNA with primers. WT band (F1+R1: 0 bp; F2+R2: 349 bp); *Tipe0*$^{-/-}$ band (F1+R1: 908 bp; F2+R2: 0 bp). (B) Genotyping PCR on mice tail genomic DNA with primers. WT band (704 bp); *Tipe1*$^{-/-}$ band (516 bp). (C) Genotyping PCR on mice tail genomic DNA with primers. WT band (852 bp); *Tipe2*$^{-/-}$ band (534 bp). (D) Quantification of *Tipe0*, *Tipe1*, and *Tipe2* mRNA expression by quantitative real-time PCR (qPCR) in thymocytes isolated from WT (n=3) and *Tipe0*$^{-/-}$*Tipe1*$^{-/-}$*Tipe2*$^{-/-}$ (n=3) mice. Data are representative of two independent experiments. Data are presented as mean ± SEM.

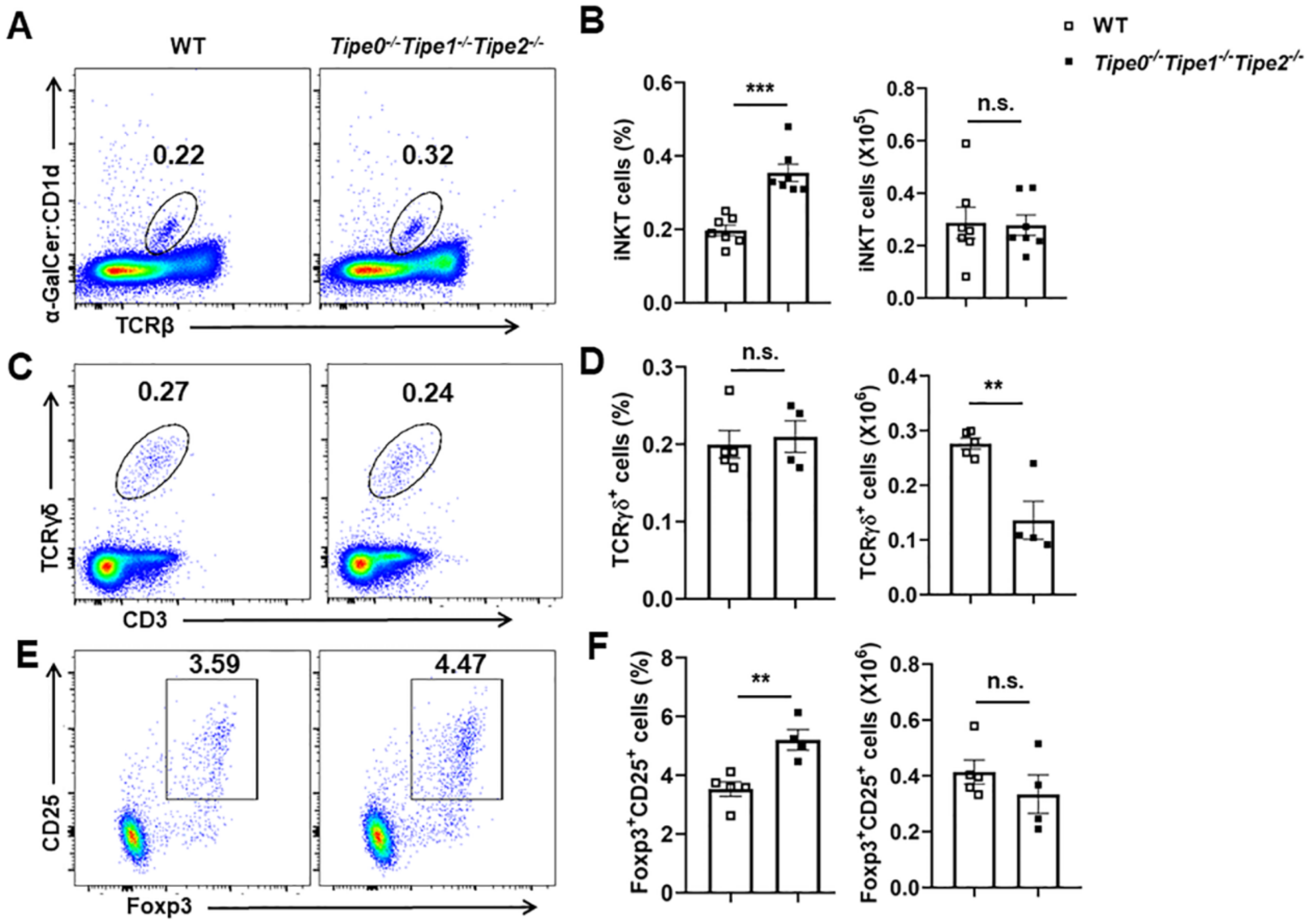

Figure S3. Thymic iNKT cells, γδ T cells, and Treg cells in WT and *Tipe0$^{-/-}$Tipe1$^{-/-}$Tipe2$^{-/-}$* mice.

(A-B) FACS analysis of iNKT cells in the thymus from WT (n=7) and *Tipe0$^{-/-}$ Tipe1$^{-/-}$Tipe2$^{-/-}$* (n=7) mice, presented as a representative FACS plot (A) and summary graphs (B). (C-D) FACS analysis of γδ T cells in the thymus from WT (n=5) and *Tipe0$^{-/-}$ Tipe1$^{-/-}$Tipe2$^{-/-}$* (n=4) mice, presented as a representative FACS plot (C) and summary graphs (D). (E-F) FACS analysis of Treg cells in the thymus from WT (n=5) and *Tipe0$^{-/-}$ Tipe1$^{-/-}$Tipe2$^{-/-}$* (n=4) mice, presented as a representative FACS plot (E) and summary graphs (F). Data are representative of at least three independent experiments. Data are presented as mean± SEM. Student's *t* test (B, D and F), $^{**}P<0.01$, $^{***}P<0.001$, n.s. non-significant.

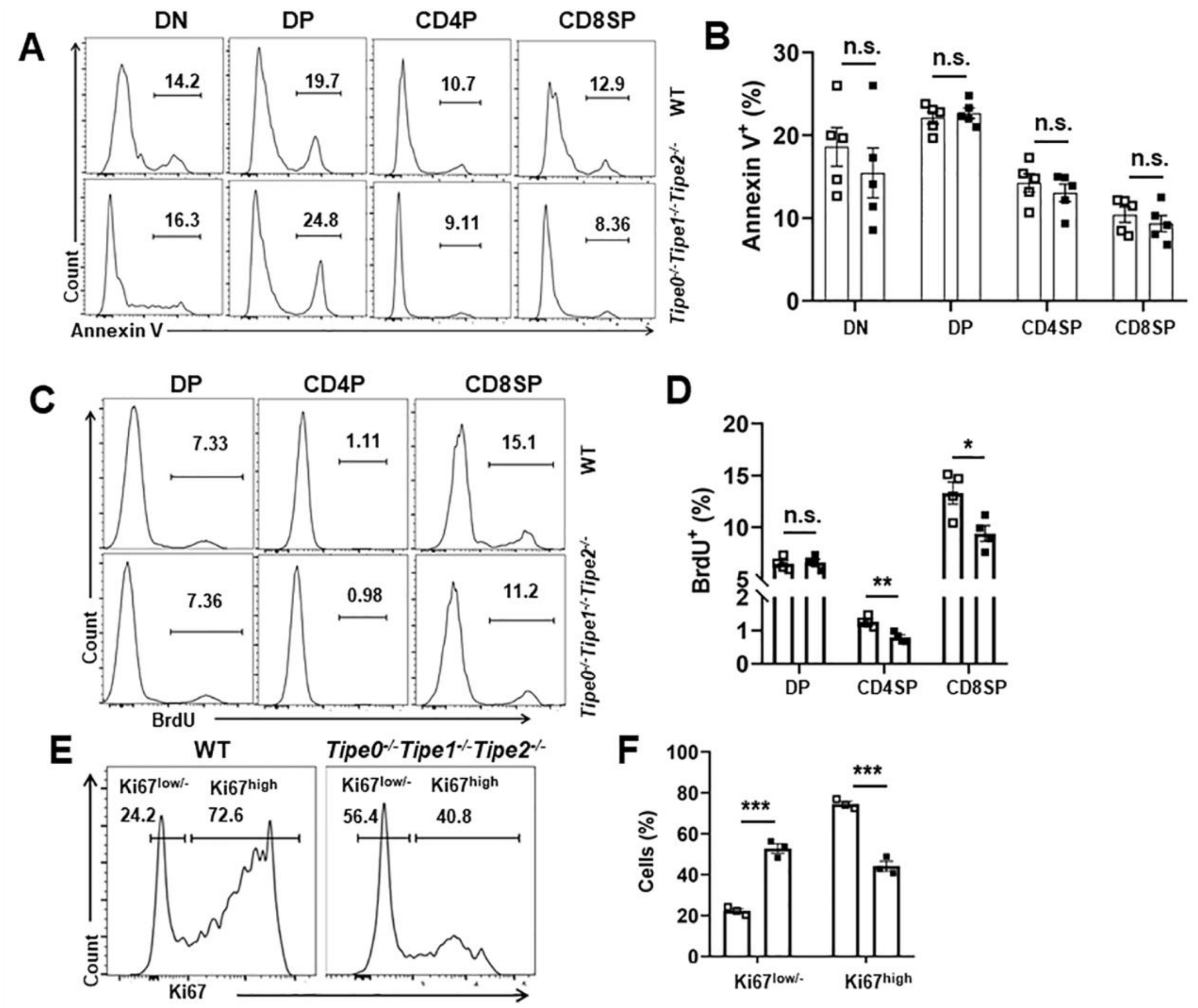

Figure S4. Effect of TNFAIP8 deletion on apoptosis and proliferation of thymocytes.

(A-B) Apoptosis in thymocyte subsets was analyzed with Annexin V staining. Representative histograms (A) show the percentages of Annexin V^+ cells in indicated subsets from WT (n=5) and *Tipe0*$^{-/-}$ *Tipe1*$^{-/-}$*Tipe2*$^{-/-}$ (n=5) mice. Frequencies of Annexin V^+ cells are shown in (B). (C) Representative histograms of BrdU incorporation in DP, CD4SP, and CD8SP thymocytes from WT (n=4) and *Tipe0*$^{-/-}$ *Tipe1*$^{-/-}$*Tipe2*$^{-/-}$ (n=4) mice at 4 hours after BrdU injection. (D) Frequencies of $BrdU^+$ cells among subsets as in (C). (E) Representative histograms of Ki67 staining in DN thymocytes from WT (n=3) and *Tipe0*$^{-/-}$ *Tipe1*$^{-/-}$*Tipe2*$^{-/-}$ (n=3) mice. (F) Frequencies of $Ki67^{low/-}$ and $Ki67^{high}$ DN cells as in (E). Data are representative of at least three independent experiments. Data are presented as mean± SEM. Student's *t* test (B, D and F), **P*<0.05, ***P*<0.01, ****P*<0.001, n.s. non-significant.

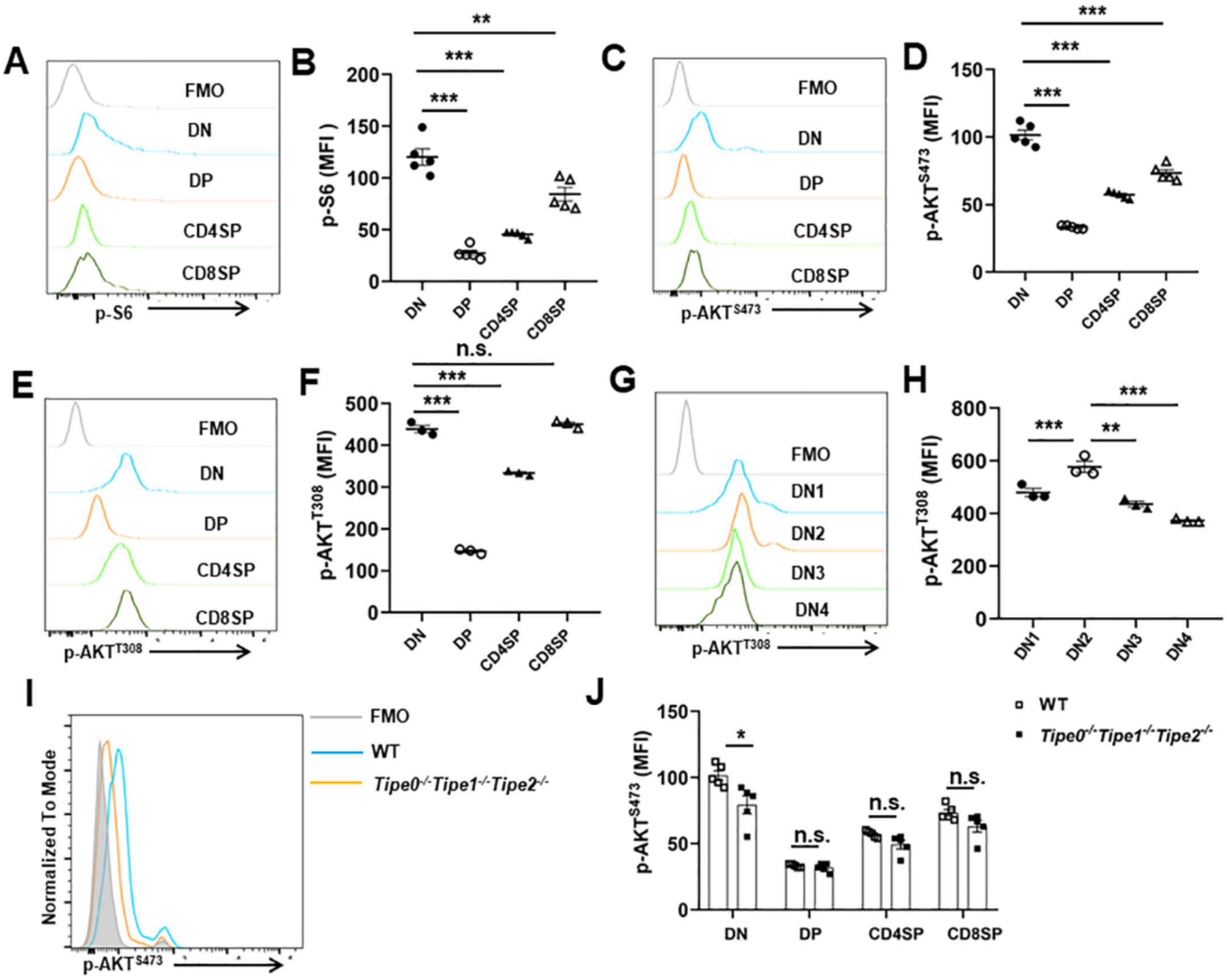

Figure S5. mTORC1 and mTORC2 activities in thymocytes.

(A-B) FACS analysis of p-S6 levels in DN, DP, CD4SP, and CD8SP from WT mice (n=5), presented as representative histograms (A) and summary graph (B). (C-D) FACS analysis of p-AKT(S473) levels in DN, DP, CD4SP, and CD8SP from WT mice (n=5), presented as representative histograms (C) and summary graph (D). (E-F) FACS analysis of p-AKT(T308) levels in DN, DP, CD4SP, and CD8SP from WT mice (n=5), presented as representative histograms (E) and summary graph (F). (G-H) FACS analysis of p-AKT(T308) levels in DN1, DN2, DN3, and DN4 from WT mice (n=5), presented as representative histograms (G) and summary graph (H). (I-J) FACS analysis of p-AKT(S473) in DN, DP, CD4SP, and CD8SP from WT (n=5) and *Tipe0$^{-/-}$Tipe1$^{-/-}$Tipe2$^{-/-}$* (n=5) mice, presented as representative plots (I) only for DN and summary graph (J) for DN, DP, CD4SP, and CD8SP. Data are representative of three independent experiments. Data are presented as mean± SEM. Student's *t* test (B, D, F, H and J), *P<0.05, **P<0.01, ***P<0.001, n.s. non-significant.

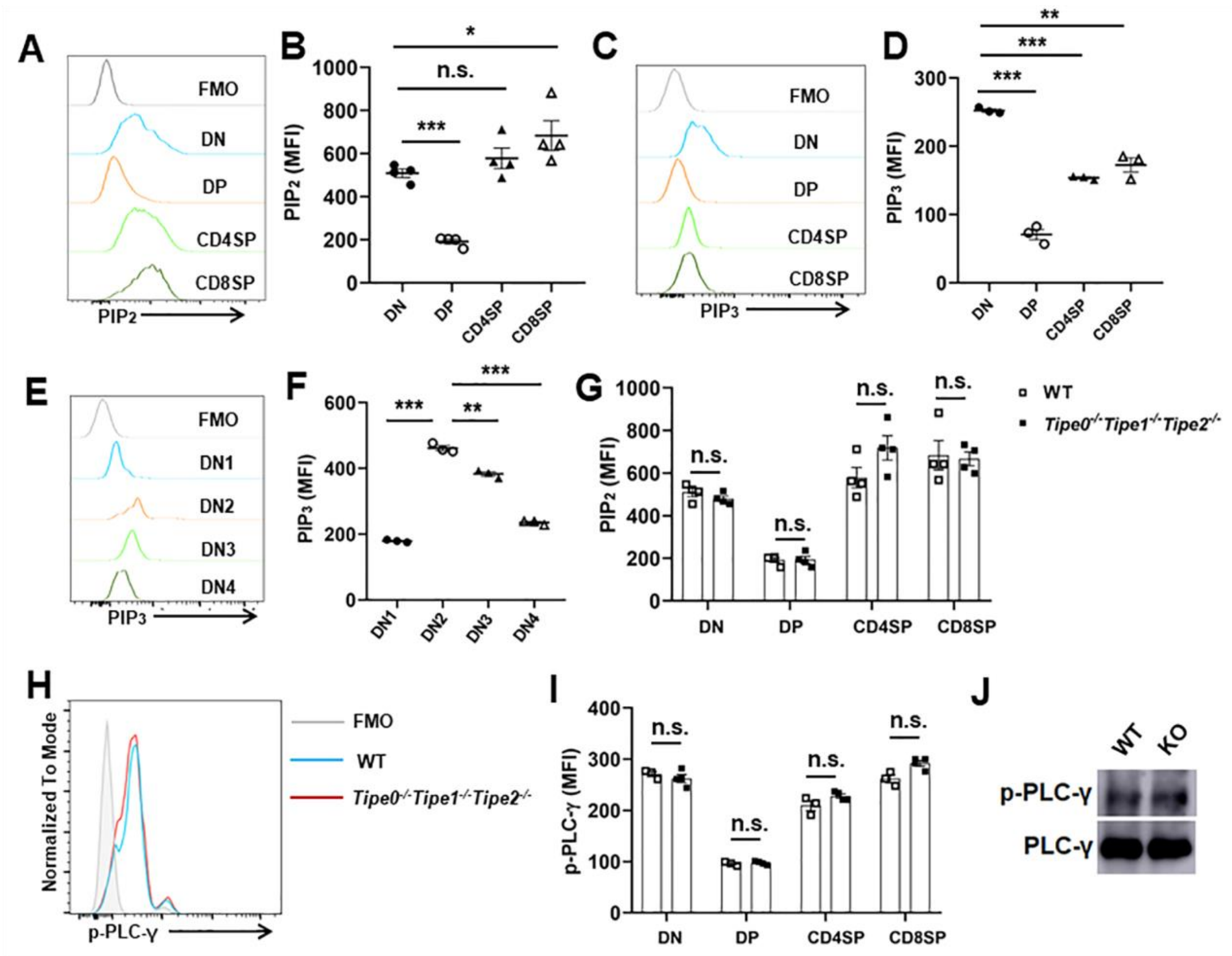

Figure S6. PIP2 and PIP3 levels in thymocytes and the effects of TNFAIP8 deletion on maintaining PIP2 levels and PLC-γ activity in thymocytes.

(A-B) FACS analysis of PIP2 levels in DN, DP, CD4SP, and CD8SP from WT mice (n=4), presented as representative histograms (A) and summary graph (B). (C-D) FACS analysis of PIP3 levels in DN, DP, CD4SP, and CD8SP from WT mice (n=3), presented as representative histograms (C) and summary graph (D). (E-F) FACS analysis of PIP3 levels in DN1, DN2, DN3, and DN4 from WT mice (n=3), presented as representative histograms (E) and summary graph (F). (G) FACS analysis of PIP2 levels in DN, DP, CD4SP, and CD8SP from WT (n=4) and *Tipe0*$^{-/-}$ *Tipe1*$^{-/-}$*Tipe2*$^{-/-}$ (n=4) mice. MFI of each subset is shown. (H-I) FACS analysis of p-PLC-γ in DN, DP, CD4SP, and CD8SP from WT (n=3) and *Tipe0*$^{-/-}$ *Tipe1*$^{-/-}$*Tipe2*$^{-/-}$ (n=3) mice, presented as representative plots (D) only for DN and summary graph (E) for DN, DP, CD4SP, and CD8SP. (J) Western blot analysis of the indicated proteins in WT and *Tipe0*$^{-/-}$ *Tipe1*$^{-/-}$*Tipe2*$^{-/-}$ DN thymocytes. Data are representative of three independent

experiments. Data are presented as mean± SEM. Student's *t* test (B, D, F, G and I), *P<0.05, **P<0.01, ***P<0.001, n.s. non-significant.

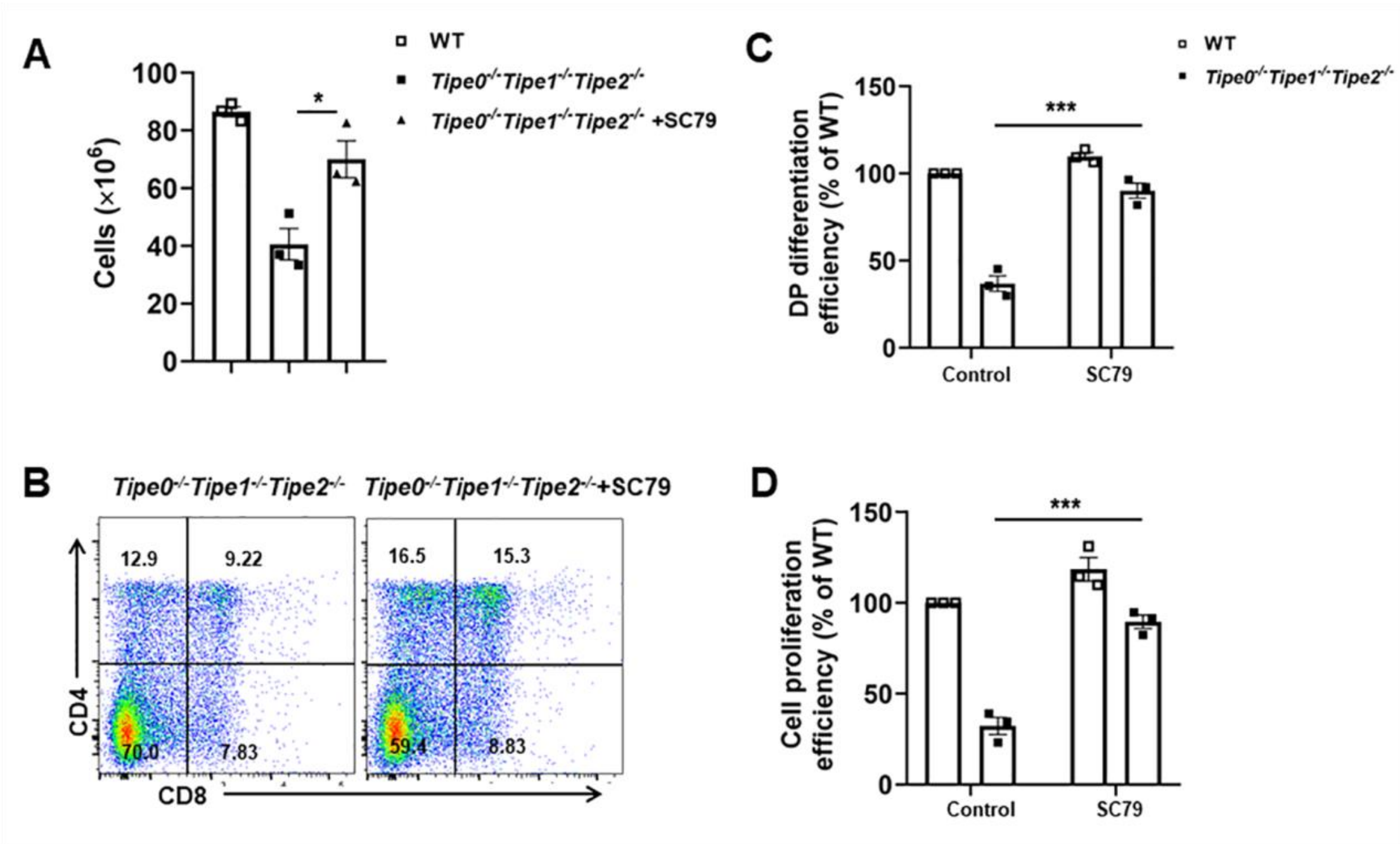

Figure S7. Active AKT is epistatic to TNFAIP8 in Notch-driven pre-T cell differentiation and proliferation.

(A) Quantification of total thymocytes from WT (n=4), *Tipe0*$^{-/-}$ *Tipe1*$^{-/-}$*Tipe2*$^{-/-}$ (n=3), and *Tipe0*$^{-/-}$ *Tipe1*$^{-/-}$*Tipe2*$^{-/-}$ (n=3) mice injected with SC79. (B) FACS analysis of the effect of SC79 on DN thymocyte differentiation purified from *Tipe0*$^{-/-}$ *Tipe1*$^{-/-}$*Tipe2*$^{-/-}$ mice. Shown is a CD4 and CD8 staining of DN cells cultured with OP9-DL1 monolayer for 6 days. (C) Purified CD4^{-}CD8^{-} DN thymocytes from WT (n=3) and *Tipe0*$^{-/-}$*Tipe1*$^{-/-}$*Tipe2*$^{-/-}$ (n=3) mice were cultured with or without SC79 on isogenic stromal cells bearing Notch ligand (OP9-DL1) in the presence of IL-7. Mean efficiency of differentiation into DP thymocytes which was normalized to WT cells grown on OP9-DL1. (D) Mean population increases, with normalization and replicates as in A. Data are representative of three independent experiments. Data are presented as mean± SEM. Student's *t* test (A, C and D), *P<0.05, ***P<0.001.

Table S1. Antibodies for flow cytometry and Western blot

Antigen	Fluorochrome	Clone	Catalog No.	Supplier
anti-CD45	PE	30-F11	103106	Biolegend
anti-CD44	FITC	IM7	103006	Biolegend
anti-CD45	eFluor 450	30-F11	115519	eBioscience
anti-CD25	PE	PC61.5.3	MA5-17817	eBioscience
anti-CD4	PE/Cyanine7	RM4-5	100528	Biolegend
anti-CD69	Brilliant Violet 421	H1.2F3	104527	Biolegend
anti-CD5	PE/Cyanine5	53-7.3	100609	Biolegend
anti-IL-7Rα	APC/Cyanine7	A7R34	135013	Biolegend
anti-CD8a	APC/Cyanine7	53-6.7	100714	Biolegend
anti-CD62L	APC	MEL-14	104411	Biolegend
anti-CD62L	FITC	MEL-14	561917	BD Pharmingen
anti-TCR β	Brilliant Violet 605	H57-597	109241	Biolegend
anti-TCR β	FITC	H57-597	109205	Biolegend
anti-CD45	eFluor 450	30-F11	48-0451-82	eBioscience
anti-CD4	FITC	GK1.5	11-0041-85	eBioscience
anti-CD8	PE	53-6.7	12-0081-82	eBioscience
anti-Actin	-	2D4H5	66009-1-Ig	Proteintech
anti-AKT1/2/3	-	ST48-09	ET1609-51	Huabio
anti-Akt (pS473)	-	D9E	4060S	CST
anti-PLCγ1	-	ARC1345	A8899	Abclonal
anti-CD16/32	-	93	101320	Biolegend
anti-TCR β	Brilliant Violet 421	H57-597 -	109229	Biolegend
Fixable Aqua Dead Cell Stain Kit	Brilliant Violet 510		L34957	Invitrogen
anti-p-PLCγ1	-	D6M9S	2821S	CST
anti-p-S6	APC	D57.2.2E	14733S	CST

anti-Akt (pS473)	Brilliant Violet 421	M89-61	562599	BD Bioscience
anti-Akt (pT308)	-	D25E6	13038S	CST
anti-Rabbit IgG	APC	-	A-21206	Invitrogen
anti-CD45.1	PerCP/Cyanine5.5	A20	110727	Biolegend
anti-CD45.2	FITC	104	11-0454-82	eBioscience
